# SCEP3 initiates synapsis and implements crossover interference in *Arabidopsis*

**DOI:** 10.1038/s41477-025-02155-x

**Published:** 2025-11-18

**Authors:** Paul J. Seear, Henry J. A. Dowling, Maja Szymańska-Lejman, Wojciech Dziegielewski, Simona Debilio, F. Chris H. Franklin, Kevin D. Corbett, Owen R. Davies, Piotr A. Ziolkowski, James D. Higgins

**Affiliations:** 1 Division of Genetics and Genome Biology, University of Leicester, Leicester, UK; 2 Laboratory of Genome Biology, Institute of Molecular Biology and Biotechnology, Adam Mickiewicz University, Poznań, Poland; 3 Institute of Bioorganic Chemistry, Polish Academy of Sciences, Poznań, Poland; 4 Wellcome Centre for Cell Biology, Institute of Cell Biology, University of Edinburgh, Edinburgh, UK; 5 School of Biosciences, University of Birmingham, Birmingham, UK; 6 Department of Cellular and Molecular Medicine, University of California, San Diego, La Jolla, CA USA; 7 Department of Molecular Biology, University of California, San Diego, La Jolla, CA USA

**Keywords:** Plant reproduction, Cell biology, Plant genetics

## Abstract

The synaptonemal complex (SC) is a meiosis-specific tripartite proteinaceous structure that regulates the number and positions of crossovers (COs). Here we characterize SCEP3, a new *Arabidopsis* SC component that is essential for CO assurance, promoting positive CO interference and preventing negative CO interference. SCEP3 localizes to the chromosome axes as numerous foci at leptotene, of which a small proportion cluster as large foci that initiate synapsis. SCEP3 then relocates to the central region of the SC as ZYP1 polymerizes. In the absence of SCEP3, homologues align but do not synapse. In the *scep3* mutants, COs increase in number towards the chromosome ends and are more likely to cluster together. *SCEP3* encodes an 801-amino-acid intrinsically disordered protein that is structurally similar to SIX6OS1 in mammals and SYP-4 in nematodes, containing phenylalanine repeats at the amino terminus and a carboxy-terminal coiled-coil, suggesting that it is a fundamentally conserved SC component across kingdoms.

## Abstract (web summary)

SCEP3 is a new synaptonemal complex protein that prevents clustering of crossovers during meiosis in *Arabidopsis*, so that every pair of homologous chromosomes receives at least one ‘obligate’ crossover.

## Main

Meiosis is a specialized cell division that produces haploid cells during sexual reproduction. In *Arabidopsis thaliana*, five pairs of homologous chromosomes recombine during meiosis I to form reciprocal crossovers (COs) that ensure balanced chromosome segregation at meiosis II, as well as creating new allelic combinations. Meiotic recombination in *A. thaliana* is initiated by ~200 programmed DNA double-strand breaks (DSBs) that result in around nine class I COs (85% of the total COs) and around one class II CO (15%)^1,2^. Class I COs ensure that every chromosome pair receives at least one obligate CO and are sensitive to CO interference (the phenomenon in which two COs are spaced further apart than expected by chance)^3,4^, whereas class II COs are insensitive to CO interference and do not maintain the obligate CO^5,6^. A complex of proteins including Spo11-1 (ref. ^7^), Spo11-2 (ref. ^8^), PRD1-3 (refs. ^9,10^), DFO1 (ref. ^11^) and MTOPVIB^12^ coordinate the formation of DSBs that are resected into single-stranded DNA overhangs by the MRN complex (MRE11 (ref. ^13^), RAD50 (ref. ^14^) and NBS1 (ref. ^15^)). RAD51 and DMC1 then coat the resected single-stranded DNA ends to mediate strand invasion, thereby promoting the pairing of chromosomes by homology searching as well as forming D-loop recombination intermediates^16–18^. D-loops are processed into double Holliday junctions by a complex of proteins including HEI10 (ref. ^19^), MER3 (refs. ^2,20^), MSH4 (ref. ^2^), MSH5 (ref. ^20^), SHOC1 (ref. ^21^), ZIP4 (ref. ^22^) and PTD^23^ that are resolved into COs by MLH1 (refs. ^24,25^) and MLH3 (ref. ^26^), although in *Arabidopsis* only ~5% of DSBs mature into COs, and the other ~95% are repaired as non-COs.

Meiotic recombination occurs in the context of the chromosome axis and the synaptonemal complex (SC). The chromosome axis is a filamentous proteinaceous structure that promotes inter-homologue DNA recombination and repair^27^. The cohesins SYN1/REC8 (refs. ^28,29^), SMC1 and SMC3 (ref. ^30^) anchor sister chromatid DNA loops, thus providing a scaffold for the meiosis-specific proteins ASY1 (ref. ^31^), ASY3 (ref. ^32^) and ASY4 (ref. ^33^) to localize and promote inter-homologue recombination in collaboration with RAD51 and DMC1. DMC1-mediated strand invasion of the homologue is required for the installation of the SC transverse filament proteins ZYP1a and ZYP1b^34^ and central element proteins SCEP1 and SCEP2 (ref. ^35^). Despite limited sequence homology, functional orthologues of several SC proteins have been identified in *Arabidopsis*, mammals and yeast (ASY1 ≡ HORMAD1/2/Hop1, ASY3 ≡ SYCP2/Red1, ASY4 ≡ SYCP3, ZYP1 ≡ SYCP1/Zip1), indicating structural conservation between kingdoms. ASY1 promotes recombination away from the telomeres in a dosage-dependent manner and maintains the fidelity of crossing over^36^. ASY1 contains a highly conserved amino-terminal HORMA domain that enables binding to the chromosome axis through its interaction with ASY3 (ref. ^32^). ASY3 is an intrinsically disordered protein that possesses a conserved carboxy-terminal coiled-coil that interacts with ASY4 to form filamentous fibres^27^. ZYP1a and ZYP1b are α-helical proteins, predicted to form coiled-coils that span the SC^34^. The ZYP1 C termini imbed into the chromosome axes (referred to as lateral elements), and the N termini localize to the central element^34^. In the absence of SC central region proteins ZYP1, SCEP1 and SCEP2, COs increase ~1.5-fold, but CO assurance is abolished^35,37,38^. This can be explained by a model in which the SC acts as a CO-inhibitory signal that emanates from a designated CO site and prevents proximal recombination sites from maturing into COs by recruiting PCH2 to remove ASY1, thereby switching DNA repair from the homologous template to the sister chromatid^39,40^. In this model, the obligate CO would be maintained if HEI10 localization is restricted in number to individual chromosomes by the SC, so that a limited pool of HEI10 protein could be distributed to all chromosomes. An alternative interpretation of these data is that the coarsening of HEI10 determines CO assurance and CO interference^41–43^.

Our previous investigation into the role of SC genes in meiotic adaptation to whole-genome duplication in *Arabidopsis lyrata* and *A. arenosa* revealed that the segregation of a serine-rich tandem duplicated (*TD*) allele of *ASY3* was associated with meiotic stability in the autotetraploids. However, variation of meiotic stability within the *ASY3* heterozygous plants carrying the *TD* allele and non-duplicated (*ND*) *ASY3* alleles could not be explained by the segregation of known *Arabidopsis* axis/SC genes. This led us to hypothesize that alleles of an unknown gene or genes influencing CO patterning were segregating in these populations that were affecting meiotic stability^44^. We therefore revisited the list of differentiated genes between diploid and autotetraploid *A*. *lyrata* and *A. arenosa*^45,46^. *SCEP3* was the only unknown gene significantly differentiated in both datasets, so we investigated it for a role in meiotic recombination. SCEP3 was also recently identified in *A. thaliana* through analysis of biotinylated ASY1 proximity-labelled proteins^47^. SCEP3 is an intrinsically disordered protein possessing a conserved C-terminal coiled-coil and an N terminus that contains phenylalanine repeats similar to the C termini of SC proteins SYP-4 in *Caenorhabditis elegans*^48^ and SIX6OS1 in mouse^49^. SCEP3 is essential for synapsis, CO assurance and CO interference and may be functionally conserved across the majority of eukaryotes.

## Results

### *SCEP3* is required for normal fertility

**Extended Data Fig. 1:**
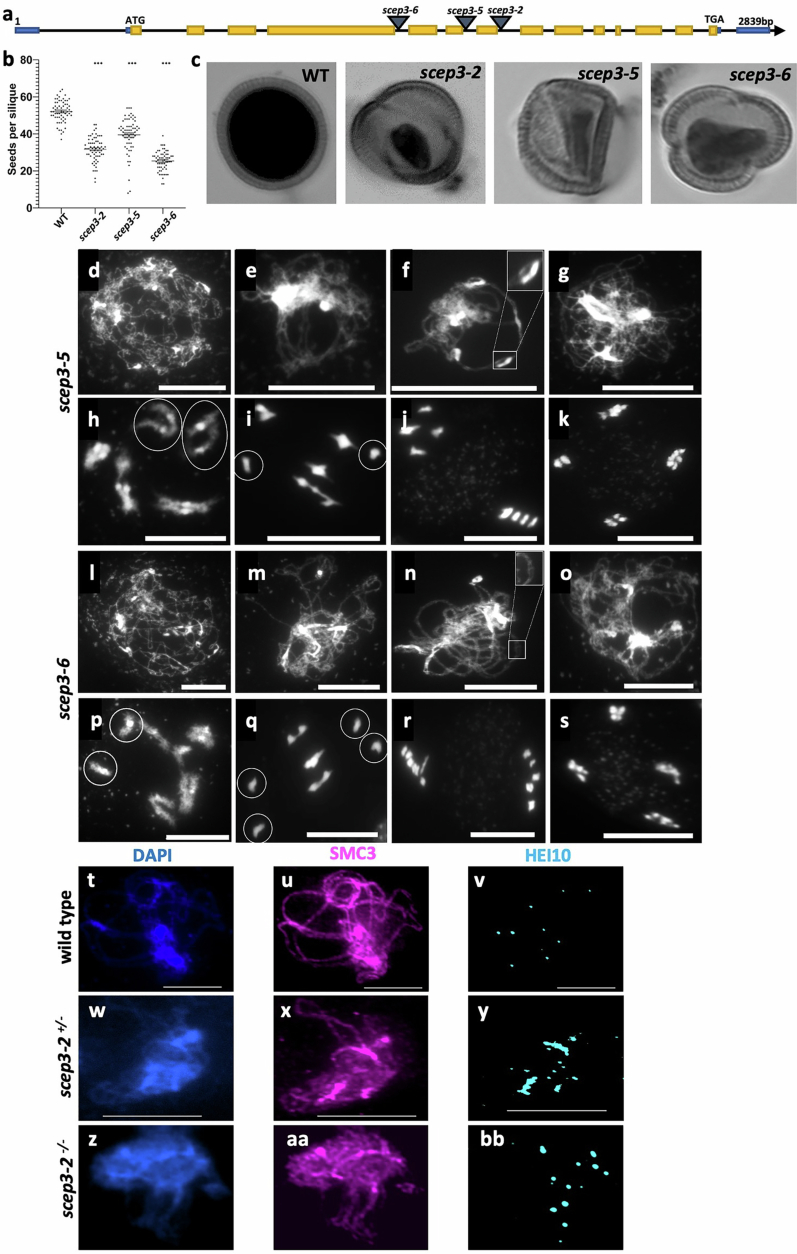
The SCEP3 gene model showing locations of T-DNA insertions and how they affect fertility, a cytological analysis of chromosome behaviour and HEI10 crossover counts. **a** The gene model of *SCEP3* is based on the cloned and sequenced transcript from the *SCEP3.5* splice variant. The untranslated regions are represented by blue boxes, the exons by yellow boxes and the black line as introns. The T-DNA insertions of the lines used here as shown with triangles. **b** Fertility was measured by comparing the numbers of seeds per silique produced in wild type and *scep3* mutants (*** represents significant differences p < 0.001 from a Chi-squared test). **c** Pollen viability was assessed by performing Alexander staining and comparing viable to non-viable pollen (examples shown). DAPI-stained meiotic chromosomes from *scep3-5* **d-k** and *scep3-6* **l-s**. Leptotene **d,l**, zygotene **e,m**, pachytene **f,n** (inserts show a gap between homologous chromosomes), diplotene **g,o**, diakinesis **h,p** (white circles highlight univalents), metaphase I **i,q** (white circle highlights univalents), metaphase II **j,r**, and telophase II **k,s**, **t-bb** Co-immunofluorescence of SMC3 (magenta) and HEI10 (cyan) on wild type **t-v**, *scep3-2*^+/−^ **w-y**, and *scep3-2*^*−/−*^ **z-bb** Arabidopsis late meiotic prophase I chromosome spreads (single channels). Scale bars = 10 µm. Source data

Fertility is significantly lower in three independent *scep3* transfer DNA (T-DNA) mutants than in the wild type (wild type, 52 ± 0.83 (mean ± s.e.m.) seeds per silique, *n* = 62; *scep3-2*, 32 ± 0.9, *n* = 64; *scep3-5*, 39 ± 1.3, *n* = 62; *scep3-6*, 25 ± 0.7, *n* = 60; *P* < 0.001; Extended Data Fig. 1). Alexander-stained viable pollen grains are also significantly reduced in *scep3* (in the wild type, 98% of 690 pollen grains were viable, compared with 91% (*n* = 605) in *scep3-2* (*χ*^2^ = 57.385, d.f. = 1, *P* < 0.001), 96% (*n* = 193) in *scep3-5* (*χ*^2^ = 15.348, d.f. = 1, *P* < 0.001) and 89% (*n* = 548) in *scep3-6* (*χ*^2^ = 14.566, d.f. = 1, *P* < 0.001); Extended Data Fig. 1), although the mean 6% reduction in pollen viability does not account for the 38% reduction in fertility, indicating that oogenesis is more severely affected than gametogenesis.

### A cytological analysis reveals meiotic defects in *scep3*

**Fig. 1:**
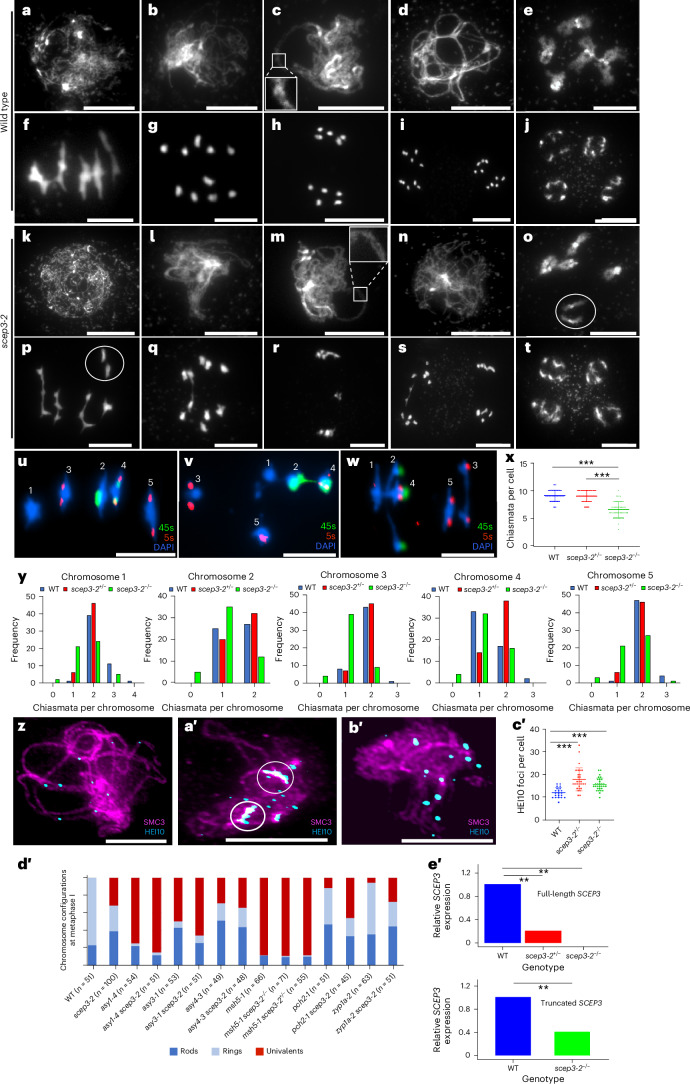
A cytological analysis of chromosome behaviour and CO counts in *scep3*. **a**–**t**, Representative DAPI-stained meiotic chromosomes from a sample of 50 wild-type (**a**–**j**) and *scep3-2* (**k**–**t**) cells. Leptotene (**a**,**k**), zygotene (**b**,**l**), pachytene (**c**,**m**) (the insets show gaps between homologous chromosomes), diplotene (**d**,**n**), diakinesis (**e**,**o**) (in **o** and **p**, white circles mark a pair of univalents), metaphase I (**f**,**p**), anaphase I (**g**,**q**), telophase I (**h**,**r**), anaphase II (**i**,**s**) and the tetrad stage (**j**,**t**) are shown. **u**–**y**, DAPI-stained meiotic metaphase I chromosomes labelled with 5S and 45S rDNA and chiasma counts from individual chromosomes. The images show the wild type (**u**), *scep3-2*^+/−^ (**v**) and *scep3-2*^−/−^ (**w**), with chromosome numbers in white. Panel **x** shows chiasma counts per cell (the data are presented as mean ± s.d., and the asterisks indicate significant differences (***P < 0.0001, two-tailed Mann–Whitney *U*-test)) for the wild type (WT) (*n* = 58 cells), *scep3-2*^+/−^ (*n* = 58 cells) and *scep3-2*^−/−^ (*n* = 52 cells). Panel **y** shows chiasma counts per chromosome. **z**–**c′**, At late meiotic prophase I, HEI10 foci mark class I CO sites in the wild type and *scep3-2*. Co-immunofluorescence of SMC3 (magenta) with HEI10 (cyan) in the wild type (**z**), *scep3-2*^+/−^ (**a′**) (the white circles highlight clusters of HEI10) and *scep3-2*^−/−^ (**b′**). Panel **c′** shows the HEI10 foci counts per cell (the data are presented as mean ± s.d.) in the wild type (*n* = 21 cells), *scep3-2*^+/−^ (*n* = 30 cells) and *scep3-2*^−/−^ (*n* = 30 cells). The asterisks indicate significant differences (***P < 0.0001, two-tailed Mann–Whitney *U*-test). **d′**, Chromosome configurations at meiotic metaphase I presented relative to the five chromosome pairs of *A. thaliana*. **e′**, qPCR shows reduced expression of full-length and truncated *SCEP3* transcripts in *scep3-2* mutants. Expression of *SCEP3* was normalized against *ACTIN* and is shown relative to the mean expression in the wild type. The data are represented as mean ± s.e.m. (*n* = 3 biological replicates, each consisting of a pool of 10 plants). Statistical significance was determined using two-tailed Welch’s *t*-tests. The asterisks indicate significant differences (for full-length *SCEP3*, *P* = 0.003 for *scep3-2*^−/−^ and *P* = 0.002 for *scep3-2*^+/−^); for truncated *SCEP3*, *P* = 0.006 for *scep3-2*^−/−^). Scale bars, 10 µm. Source data

To determine the cause of *scep3* fertility defects, a cytological analysis was performed on pollen mother cells (Fig. 1a–t and Extended Data Fig. 1). At leptotene, DAPI-stained chromosomes in *scep3* appeared indistinguishable from those in the wild type (Fig. 1a,k and Extended Data Fig. 1), but at zygotene and pachytene, large gaps between aligned chromosomes were observed in *scep3* (Fig. 1b,c,l,m and Extended Data Fig. 1). Diplotene chromosomes appeared similar in both the wild type and *scep3* mutants (Fig. 1d,n and Extended Data Fig. 1), but univalents were observed in *scep3* at diakinesis and metaphase I (Fig. 1e,f,o,p and Extended Data Fig. 1). In the wild type, no univalents were observed (*n* = 51), but in *scep3-2*, 1.9 ± 0.2 (mean ± s.e.m.) univalent pairs per cell (*n* = 100) were observed, with 1.3 ± 0.2 (*n* = 64) in *scep3-5* and 1.9 ± 0.2 (*n* = 51) in *scep3-6*. In addition, lagging chromosomes connected by chromatin bridges were observed at anaphase I and II in *scep3* (*scep3-2*, 0.7 bridges per cell, *n* = 7; *scep3-5*, 0.3, *n* = 9; *scep3-6*, 2.3, *n* = 3), but not in the wild type (*n* = 5) (Fig. 1g–i,q–s). At the tetrad stage, four haploid nuclei formed in both the wild type and *scep3* (Fig. 1j,t and Extended Data Fig. 1).

Fluorescence in situ hybridization (FISH) was performed on *scep3-2* meiotic metaphase I chromosome spreads with 45S and 5S rDNA probes to determine chiasma frequency and distribution (Fig. 1u–w). The mean numbers of chiasmata per cell in the wild type (9.1 ± 0.01, *n* = 58) and in *scep3-2*^+/−^ (9.0 ± 0.01, *n* = 58) are significantly higher than in *scep3-2*^−/−^ (6.6 ± 0.03, *n* = 58) (*P* = 3.0 × 10^−14^ and *P* = 4.4 × 10^−13^, respectively, Mann–Whitney *U*-test), but not significantly different between the wild type and *scep3-2*^+/−^ (*P* = 0.8) (Fig. 1x). It is worth noting that *scep3-2*^+/−^ metaphase I chromosomes are extremely condensed (~40% smaller than those in either the wild type (*P* = 2.3 × 10^−7^) or *scep3-2*^−/−^ (*P* = 3.5 × 10^−10^); Supplementary Table 1) and may conceal multiple COs that are spaced apart in addition to rare chromatin bridges as shown in Fig. 1v. Univalents are observed for each of the 1–5 chromosome pairs in *scep3-2*^−/−^, indicating loss of CO assurance, but not in the wild type or *scep3-2*^+/−^ (Fig. 1y). Chiasmata in chromosomes 1–3 and 5 are significantly reduced in *scep3-2*^−/−^ compared with the wild type (*P* < 0.001), but not in chromosome 4 (*P* = 0.214) (Fig. 1y). These data indicate that chiasmata are not evenly distributed between chromosomes, thereby resulting in loss of CO assurance.

Immunolocalization of HEI10 during late prophase I was performed on the wild type and *scep3-2* mutants as a marker for class I COs (Fig. 1z–c′ and Extended Data Fig. 1). HEI10 foci are significantly more numerous in *scep3-2*^−/−^ (15.8 ± 0.5, *n* = 30) than in the wild type (12.2 ± 0.5, *n* = 21) (*P* < 0.0001) (Fig. 1c′). Surprisingly, HEI10 foci in *scep3-2*^+/−^ (18.0 ± 0.9, *n* = 30) are also significantly more numerous than in the wild type (*P* < 0.0001) and cluster together, although the staging may be less accurate in *scep3-2*^+/−^ (Fig. 1a′–c′).

Metaphase I chromosome configurations were determined for *scep3-2*^−/−^ in meiotic mutant backgrounds that affect CO patterning. Chiasmata are significantly reduced in *asy1* *scep3-2* (1.5 ± 0.1, *n* = 51) compared with *asy1* (2.3 ± 0.2, *n* = 54, *P* = 0.0001), suggesting that SCEP3 promotes CO formation with asynaptic chromosomes (Fig. 1d′ and Supplementary Table 2). Chiasmata are also significantly reduced in *pch2* *scep3-2* (5.0 ± 0.2, *n* = 45) compared with either *pch2* (6.9 ± 0.2, *n* = 51) or *scep3-2* (5.9 ± 0.1, *n* = 100) (*P* = 3.3 × 10^−9^ and *P* = 0.0008, respectively), indicating codependency of these two proteins (Fig. 1d′). Univalents are significantly more numerous in *asy3* *scep3-2* (4.9 ± 0.3, *n* = 51) than in both *asy3* (3.3 ± 0.3, *n* = 53) and *scep3-2* (1.9 ± 0.2, *n* = 100) mutants (*P* = 0.0005 and *P* = 6.6 × 10^−15^, respectively), but chiasma frequency is unaffected, implying a change in CO positioning. Chiasmata in *asy4* *scep3-2* (5.3 ± 0.2, *n* = 48) are significantly reduced compared with *scep3-2* (5.9 ± 0.1, *n* = 100) (*P* = 0.049), but not compared with *asy4* (5.5 ± 0.2, *n* = 49) (*P* = 0.68), suggesting that SCEP3 functions downstream of ASY4 (Fig. 1d′). There are no significant changes in chiasmata in *zyp1* *scep3-2* (6.0 ± 0.2, *n* = 51) compared to *scep3-2* (5.9 ± 0.1, *n* = 100) (*P* = 0.63), but there is a significant reduction when compared with *zyp1* (8.2 ± 0.2, *n* = 63) (*P* = 9.7 × 10^−12^), suggesting that SCEP3 functions upstream of ZYP1 (Fig. 1d′). Chiasmata in *msh5* (1.1 ± 0.1, *n* = 66) are not significantly reduced when compared to *msh5* *scep3-2*^−/−^ (1.1 ± 0.1, *n* = 71) or *msh5* *scep3-2*^+/−^ (1.2 ± 0.1, *n* = 55; *P* = 0.93 and *P* = 0.67, respectively) (Fig. 1d′), indicating that SCEP3 is not directly required for promoting class II COs. In addition, the chromatin bridges observed in *scep3-2*^−/−^ are absent in *msh5* *scep3-2*^−/−^, consistent with a failure to repair unresolved class I CO recombination intermediates and/or inter-chromosomal telomeric connections^50^.

Quantitative PCR (qPCR) analysis revealed that full-length *SCEP3* transcripts are significantly reduced in *scep3-2*^−/−^ (−99.8% ± 0.1% (mean ± s.e.m.), *n* = 3 pools of 10) (Welch’s *t*-test, *P* = 0.003) and *scep3*^+/−^ (−79.0% ± 0.7%, *n* = 3 pools of 10) (*P* = 0.002) relative to the wild type (Fig. 1e′). There is also a significant reduction in the expression of truncated *SCEP3* transcripts upstream of the T-DNA in *scep3-2*^−/−^ (−58.5% ± 1.2%, *n* = 3 pools of 10, *P* = 0.006; Fig. 1e′).

### SCEP3 localizes to the axis and SC

**Fig. 2:**
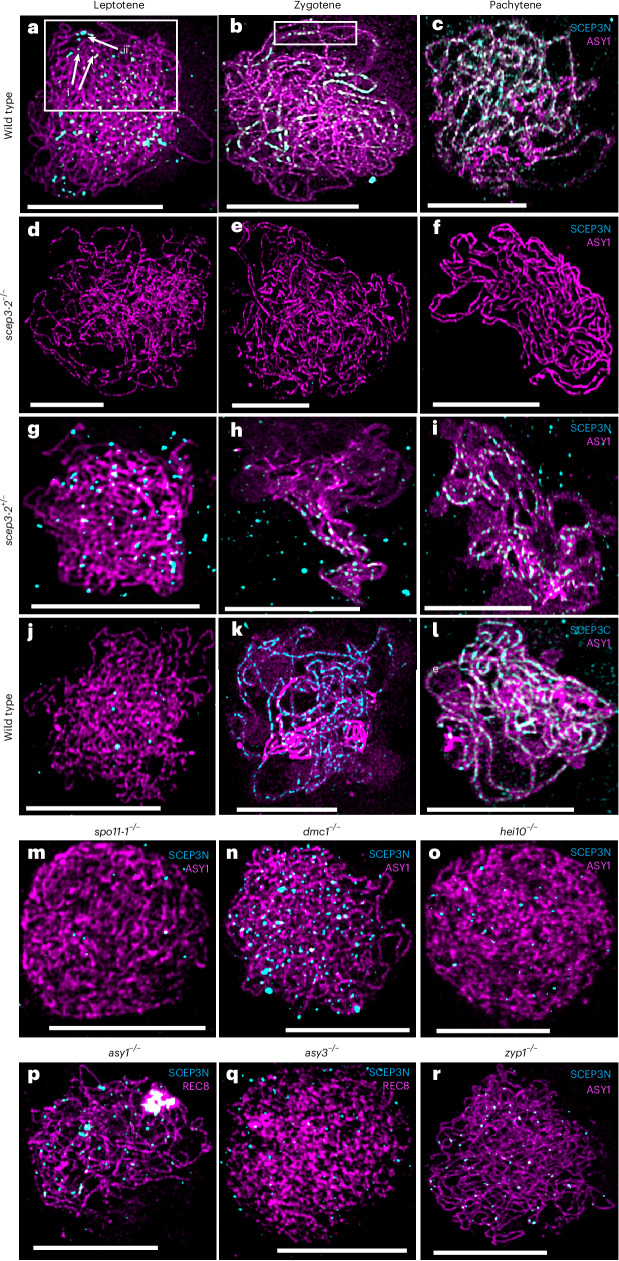
SCEP3 localizes to meiotic chromosomes at prophase I. **a**–**i**, Co-immunofluorescence of SCEP3N antibody (cyan) with ASY1 (magenta) in wild-type (**a**–**c**), *scep3-2*^−/−^ (**d**–**f**) and *scep3-2*^+/−^ (**g**–**i**) *Arabidopsis* meiotic prophase I chromosome spreads at leptotene, zygotene and pachytene. The arrows in the white box in **a** show small (i) and large (ii) foci, and SCEP3 foci localized at the SC central element preceding a synaptic fork are indicated by the white box in **b**. **j**–**l**, Co-immunofluorescence of SCEP3C antibody (cyan) with ASY1 (magenta) in wild-type *Arabidopsis* meiotic prophase I chromosome spreads at leptotene (**j**), zygotene (**k**) and pachytene (**l**). **m**–**r**, Immunolocalization of SCEP3 to recombination and SC protein mutants varies in foci number. Co-immunofluorescence of ASY1 or REC8 (magenta) with SCEP3N (cyan) in *spo11-1*, *dmc1*, *hei10*, *asy1*, *asy3* and *zyp1* homozygous mutant meiotic prophase I chromosome spreads is shown. Representative images are shown from a sample of 50 cells from each genotype. DNA is stained with DAPI (blue), apart from *hei10*. Scale bars, 10 µm.

**Fig. 3:**
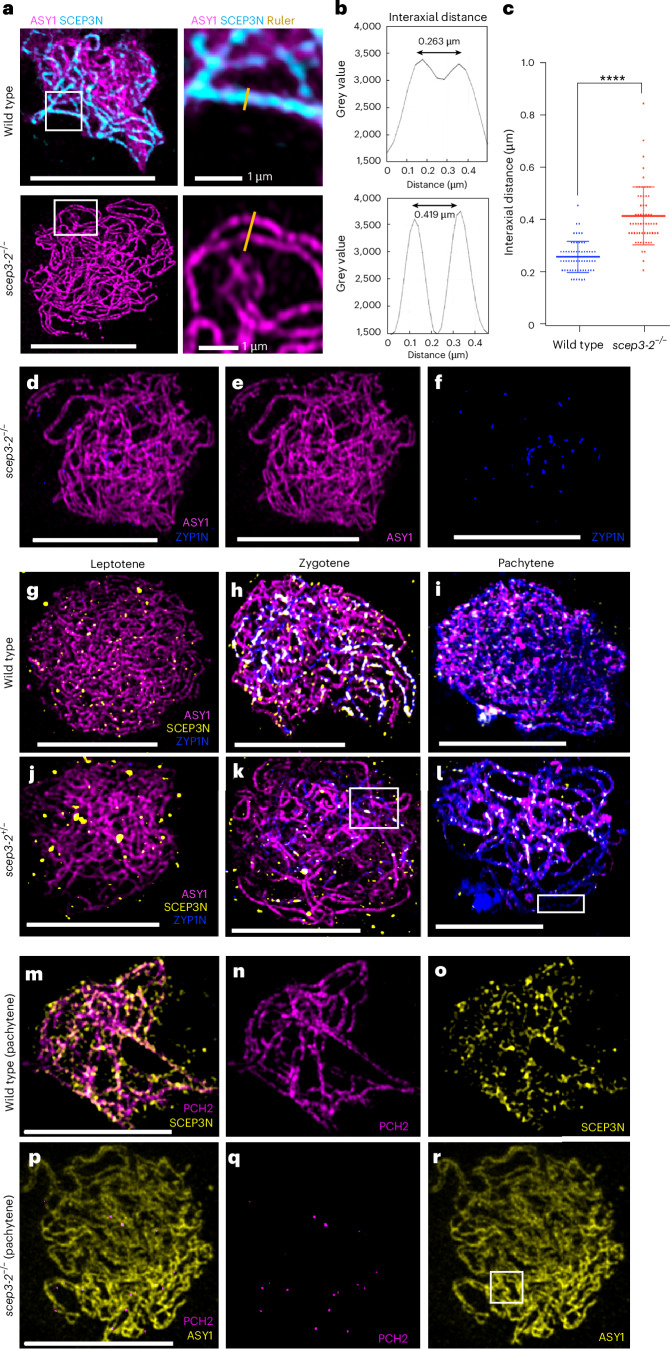
Interaxial distances measured at pachytene in the wild type and at the pachytene-like stage in the asynaptic *scep3*^−/−^ mutant. **a**, Immunolocalization of chromosome axis protein ASY1 (magenta) and SCEP3 (cyan) in the wild type and *scep3*^−/−^ showing an example of axis width measurement sampling (yellow transect in the white inset box). Representative images are shown from a sample of three cells from each genotype. Scale bars, 10 µm (left). **b**, Profile plots of examples from the interaxial measurements showing inter-peak distance. **c**, Comparison of interaxial distances in the wild type and *scep3-2* (the data are presented as mean ± s.d., *n* = 3 cells, 20 measurements taken from each cell). The asterisks indicate significant differences (****P < 0.0001, two-tailed Mann–Whitney *U*-test). **d**–**r**, ZYP1 localized as foci during zygotene in *scep3*^−/−^ (**d**–**f**), while in *scep3-2*^+/−^ ZYP1 polymerization completes but SCEP3 loading is intermittent (**g**–**l**). PCH2 co-localizes with SCEP3N during pachytene in wild type, while in *scep3*^−^^/^^−^ only a few PCH2 foci are observed (**m**–**r**). Panels **d**–**f** show co-immunofluorescence of ZYP1N (blue) with ASY1 (magenta) on *scep3*^−/−^ meiotic prophase I chromosomes at zygotene. Panels **g**–**l** show co-immunofluorescence of SCEP3N (yellow) and ZYP1 (blue) with ASY1 (magenta) on wild type (**g**–**i**) and *scep3-2*^+/−^ (**j**–**l**) meiotic prophase I chromosome spreads at leptotene, zygotene and pachytene. The white boxes in **k** and **l** highlight the intermittent SCEP3 signal relative to the complete ZYP1 signal. Panels **m**–**r** show co-immunofluorescence of SCEP3N and ASY1 (yellow) with PCH2 (magenta) in wild type (**m**–**o**) and *scep3-2*^−/−^ (**p**–**r**) meiotic prophase I chromosome spreads at leptotene, zygotene and pachytene. Intermittent PCH2 and SCEP3 signal in *scep3-2*^−/−^ can be observed in the white box in **r**. Representative images are shown from a sample of 50 cells from each genotype. Scale bars, 10 µm. Source data

**Extended Data Fig. 2:**
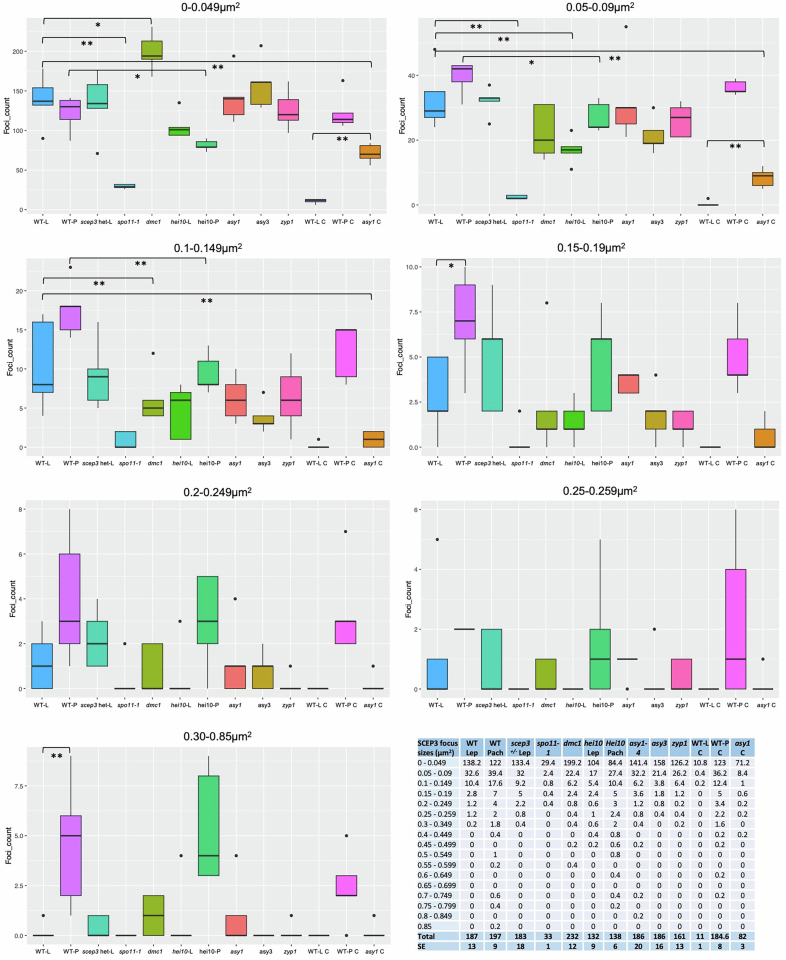
SCEP3 foci increase in size from leptotene to pachytene in wild type, small SCEP3 foci are significantly reduced in *spo11-1* and *hei10* but significantly increased in *dmc1* mutants while SCEP3 C terminus antibody observed in *asy1* but not in WT leptotene indicating masking of SCEP3 C terminus by ASY1. Boxplots represent SCEP3 foci counts in cells of wild type leptotene and pachytene, in addition to *asy1*, *asy3*, *dmc1*, *hei10, scep3*^*+/−*^, *spo11-1* and *zyp1* cells (n = 5). Leptotene only shown for asynaptic mutants (*asy1*, *asy3*, *dmc1, zyp1*), for others, L = leptotene, P = pachytene. All SCEP3 foci represent immunofluorescence with SCEP3 N terminus antibody, except for those indicated with’C’ when the SCEP3 C terminus antibody was used. Statistical significance determined by Mann Whitney U test (**, p < 0.01; *, p < 0.05).

**Extended Data Fig. 3:**
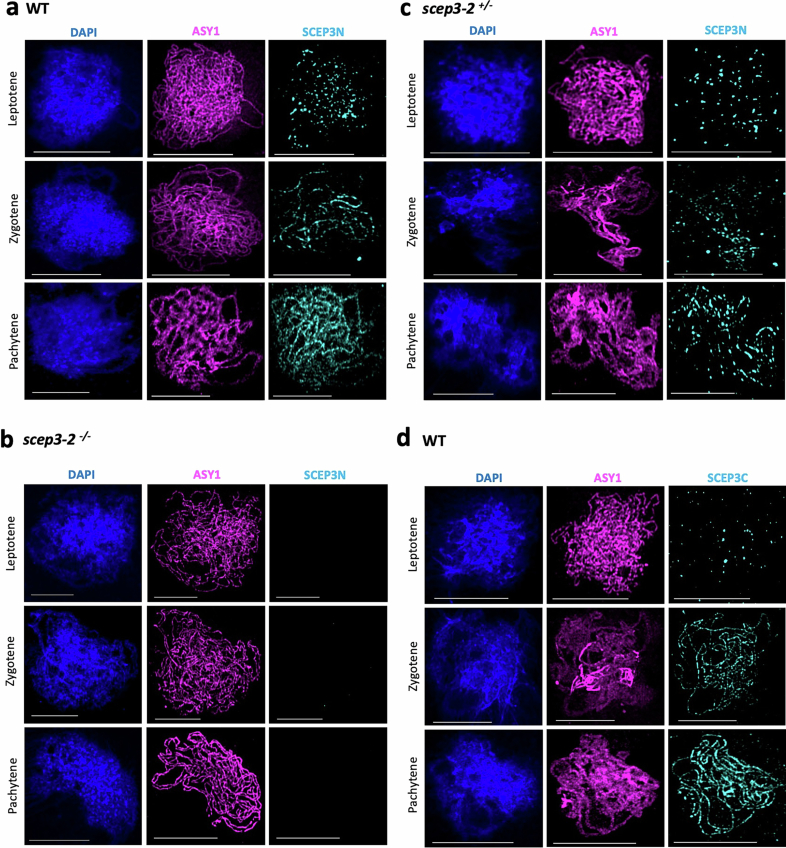
SCEP3 localises to meiotic chromosomes at prophase I in wild type and as short stretches of discontinuous foci in *scep3-2 +/-* (separate channels). Co-immunofluorescence of the N terminus (**a**-**c**) and **C** terminus (**d**) of SCEP3 (cyan) with ASY1 (magenta) on wild type (**a**, **d**), *scep3-2*^*−/−*^ (**b**) and *scep3-2*^*+/−*^ (**c**) Arabidopsis meiotic prophase I chromosome spreads at leptotene, zygotene and pachytene. DNA is stained with DAPI (blue). Scale bars = 10 µm.

**Extended Data Fig. 4:**
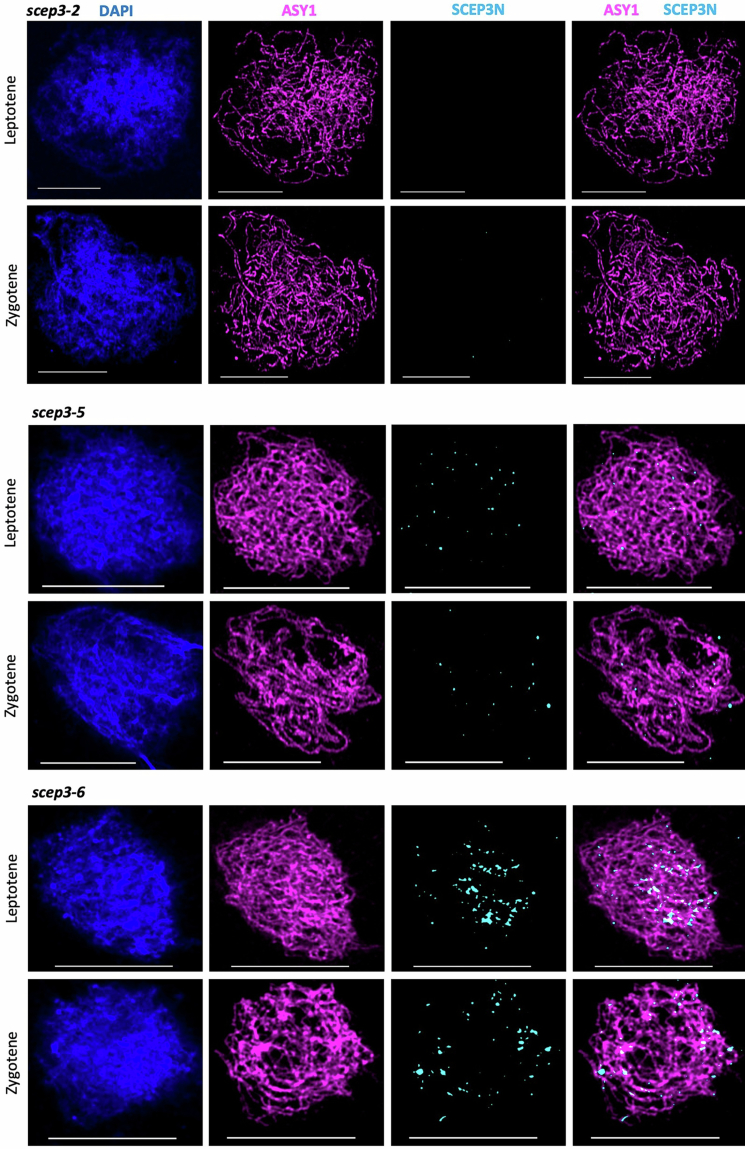
SCEP3 does not localise to meiotic chromosomes at prophase I in *scep3-2*^−/−^, while a small amount of SCEP3 protein is detected in *scep3-5*^−/−^ and *scep3-6*^−/−^. Co-immunofluorescence of SCEP3 N terminus (cyan) with ASY1 (magenta) on *scep3-2*^−/−^, *scep3-5*^−/−^ and *scep3-6*^−/−^ Arabidopsis meiotic prophase I chromosome spreads at leptotene and zygotene. DNA is stained with DAPI (blue). Scale bars = 10 µm.

**Extended Data Fig. 5:**
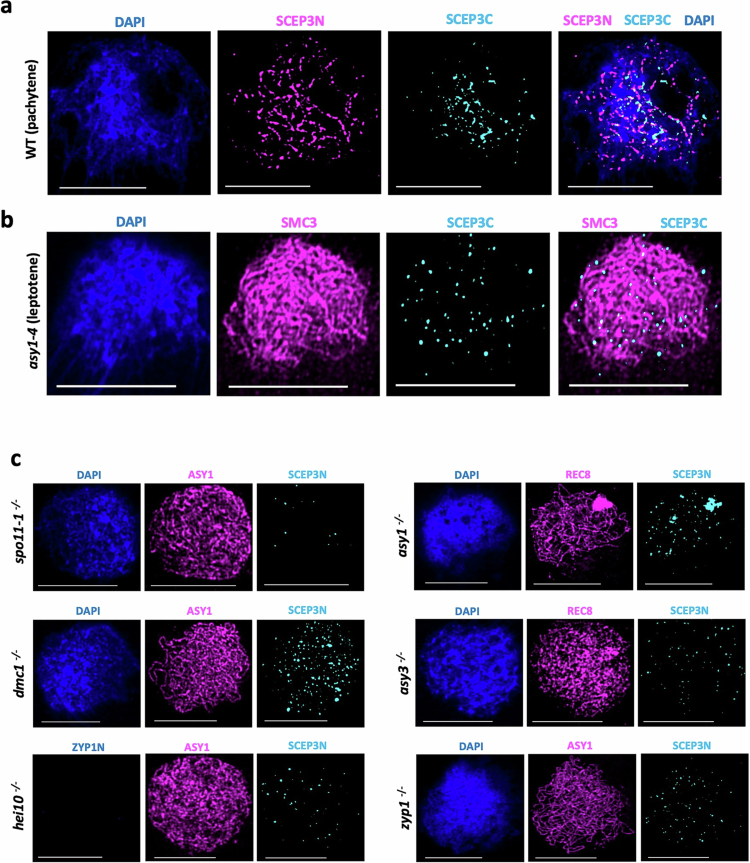
Immunolocalisation of SCEP3 to SC protein mutants varies in foci number (separate channels) and ASY1 masks SCEP3C antigen in wild type Arabidopsis at leptotene. **a** Co-immunofluorescence of SCEP3 N terminal (magenta) with SCEP3 C terminal (cyan) on wild type Arabidopsis meiotic prophase I chromosome spreads at pachytene. **b** Co-immunofluorescence of SCEP3 C terminus (cyan) with SMC3 (magenta) on *asy1-4*^*−/−*^ Arabidopsis meiotic prophase I chromosome spreads at leptotene. **c** Co-immunofluorescence of ASY1 or REC8 (magenta) with SCEP3N (cyan) on *spo11-1, dmc1, hei10, asy1, asy3* and *zyp1* homozygous mutant meiotic prophase I chromosome spreads. DNA is stained with DAPI (blue), apart from *hei10*. Scale bars = 10 µm.

Immunolocalization of antibodies raised against the N and C termini of SCEP3 (SCEP3N and SCEP3C) was performed on meiotic prophase I chromosomes. In the wild type, 187 ± 13 (mean ± s.e.m., *n* = 5) SCEP3N foci ranging in size from 0.001 to 0.35 µm^2^ were observed on the chromosome axes during leptotene (Fig. 2a and Extended Data Figs. 2 and 3). At zygotene, SCEP3N foci translocated from the axis to the central region of the SC and transitioned into larger foci (up to 0.85 µm^2^), where they persisted during pachytene (Fig. 2b,c and Extended Data Figs. 2 and 3). SCEP3N did not localize in the *scep3-2*^−/−^ mutant (Fig. 2d–f and Extended Data Figs. 2 and 3), but a small amount of residual protein was detected in *scep3-5*^−/−^ and *scep3-6*^−/−^ (Extended Data Fig. 4). At leptotene, 183 ± 18 (*n* = 5) SCEP3N foci ranging in size from 0.001 to 0.35 µm^2^ were observed in *scep3-2*^+/−^ (Fig. 2g and Extended Data Figs. 2 and 3), with short clusters localizing to the SC central region at zygotene that developed into discontinuous stretches at pachytene (Fig. 2h,i and Extended Data Fig. 3). SCEP3N foci were not confined to individual axes/SC but were often observed physically connecting disparate regions (Figs. 2a and 3m–o). SCEP3C also localized to the SC central region at zygotene and pachytene (197 ± 9, *n* = 5), overlapping with SCEP3N (Fig. 2j–l and Extended Data Figs. 3 and 5). The SCEP3C signal was lower than that of SCEP3N at leptotene but was higher in the *asy1* mutant than in the wild type, indicating that ASY1 may mask the C terminus antigen (Fig. 2j and Extended Data Fig. 5).

### SCEP3 recruitment is dependent on Spo11

To gain insight into factors controlling the recruitment of SCEP3 to meiotic chromosomes, SCEP3N was immunolocalized to recombination and SC mutants (Fig. 2m–r and Extended Data Figs. 2 and 5). At leptotene, SCEP3N foci were significantly reduced in the *spo11-1* mutant when compared with the wild type (33 ± 1 (mean ± s.e.m.), *n* = 5 versus 187 ± 13, *n* = 5; *P* = 0.01), indicating that ~84% of SCEP3 foci may localize to DSB sites. In the *dmc1* mutant, SCEP3N foci numbers were significantly higher than in the wild type (232 ± 12, *n* = 5 versus 187 ± 13, *n* = 5; *P* = 0.01), but the majority were <0.05 µm^2^, and very few transitioned into larger foci. In addition, SCEP3 foci were significantly reduced in the *hei10* mutant (132 ± 9, *n* = 5 at leptotene and 138 ± 6, *n* = 5 at pachytene), although large foci were observed at pachytene, consistent with the wild type. SCEP3 foci numbers and sizes were indistinguishable at leptotene between the wild type and the *asy1* and *asy3* mutants, although numbers were reduced in the *zyp1* mutant. These data indicate that SCEP3 localizes to DSB sites during leptotene but hyper-accumulates as small foci in the absence of DMC1-mediated homologue engagement, which may be influenced by HEI10 and ZYP1.

### SCEP3 initiates SC formation

**Extended Data Fig. 6:**
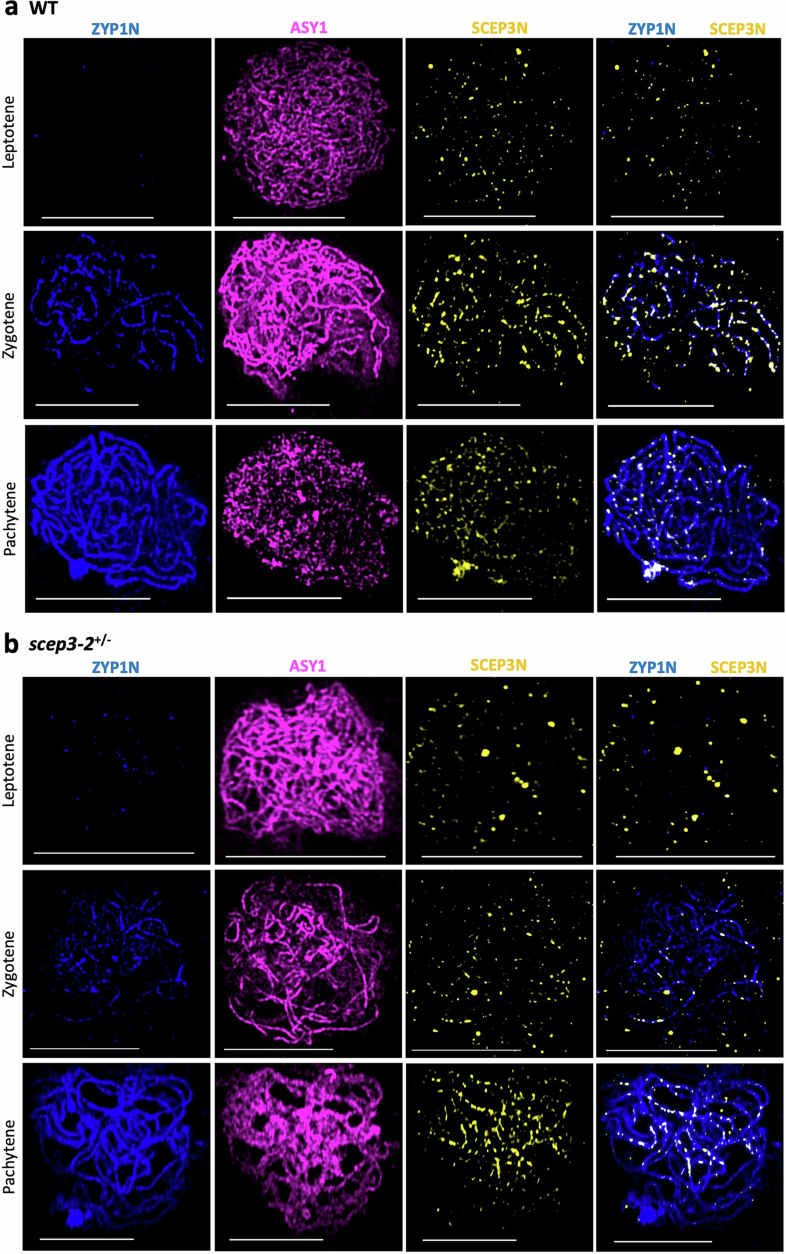
In the *scep3-2* heterozygous mutant ZYP1 signal is complete while SCEP3 is intermittent (separate channels and SCEP3N/ZYP1N merge). Co-immunofluorescence of SCEP3 N terminus (yellow) and ZYP1 (blue) with ASY1 (magenta) on wild type (**a**) and *scep3-2*^+/−^ (**b**) mutant meiotic prophase I chromosome spreads at leptotene, zygotene and pachytene. Scale bars = 10 µm.

**Extended Data Fig. 7:**
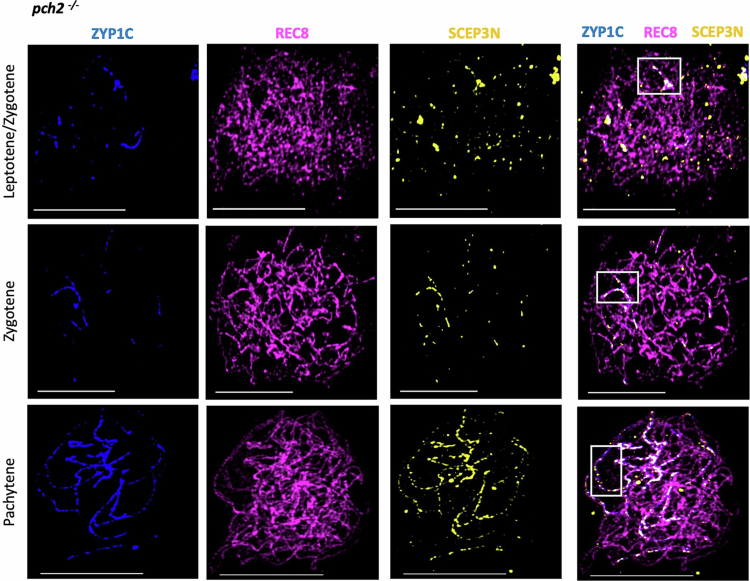
In the *pch2* mutant short stretches of ZYP1 co-localise with evenly distributed SCEP3 foci. Co-immunofluorescence of SCEP3 N terminal antibody (yellow) with ZYP1 (blue) and REC8 (magenta) on *pch2*^*−/−*^ mutant meiotic prophase I chromosome spreads at leptotene/zygotene, zygotene and pachytene. The white boxes within each image indicate evenly distributed SCEP3 foci co-localising with short ZYP1 stretches. Scale bars = 10 µm.

The mean distance between co-aligned asynapsed chromosome axes is 263 ± 8 nm in the wild type (mean ± s.e.m., *n* = 60) and 419 ± 14 nm in *scep3-2*^−/−^ (*n* = 60; *P* < 0.001; Fig. 3a–c). In *scep3-2*^−/−^, ZYP1 loaded as 28 ± 1 ZYP1C foci (*n* = 5) and 33 ± 1 ZYP1N foci (*n* = 5), of which 22 colocalized (75%) and may represent synapsis initiation centres (SICs) that are unable to extend (Fig. 3d–f, Supplementary Tables 3 and 4 and Supplementary Information). Even though ZYP1 fully polymerizes in *scep3-2*^+/−^, SCEP3N is absent in some long stretches but colocalizes with hyper-abundant residual ASY1 protein when present (Fig. 3g–l and Extended Data Fig. 6). At pachytene, SCEP3N colocalizes with PCH2, but PCH2 forms only a small number of foci in *scep3-2*^−/−^ (Fig. 3m–r). In *pch2*, short stretches of ZYP1 are observed that colocalize with evenly distributed SCEP3 foci (Extended Data Fig. 7). This indicates that SCEP3-mediated synapsis initiation does not rely on PCH2.

### SCEP3 is conserved throughout the plant kingdom

**Fig. 4:**
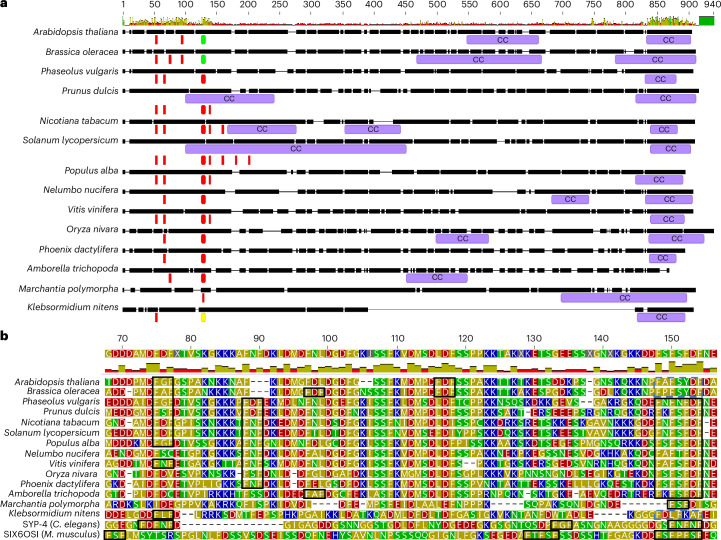
Structural prediction of SCEP3 protein highlighting conserved regions and motifs. **a**, Alignment of full-length SCEP3 protein from plant orthologues showing predicted coiled-coil domains and phenylalanine repeat motifs. Coiled-coils (CC) are annotated in purple boxes; FxF, FxFxF and FxFxFxF are highlighted by red boxes, with FxxFxxF and FxFxxxF indicated by yellow and green boxes, respectively. Amino acid number is shown at the top of the alignment above a histogram showing the level of similarity between sequences. **b**, Alignment of the N termini of SCEP3 plant orthologues with the C termini of SYP-4 (*C. elegans*) and SIX6OS1 (*Mus musculus*). FxF, FxFxF and FxFxFxF repeat motifs are indicated by black boxes, while similar FxFxxxF and FxxFxxF motifs are indicated by purple dashed boxes. The consensus sequence above the alignment is annotated with amino acid number from the N terminus along with a histogram indicating the level of conservation. Amino acid groups: red = acidic, blue = basic, green = polar uncharged, gold = hydrophobic.

**Extended Data Fig. 8:**
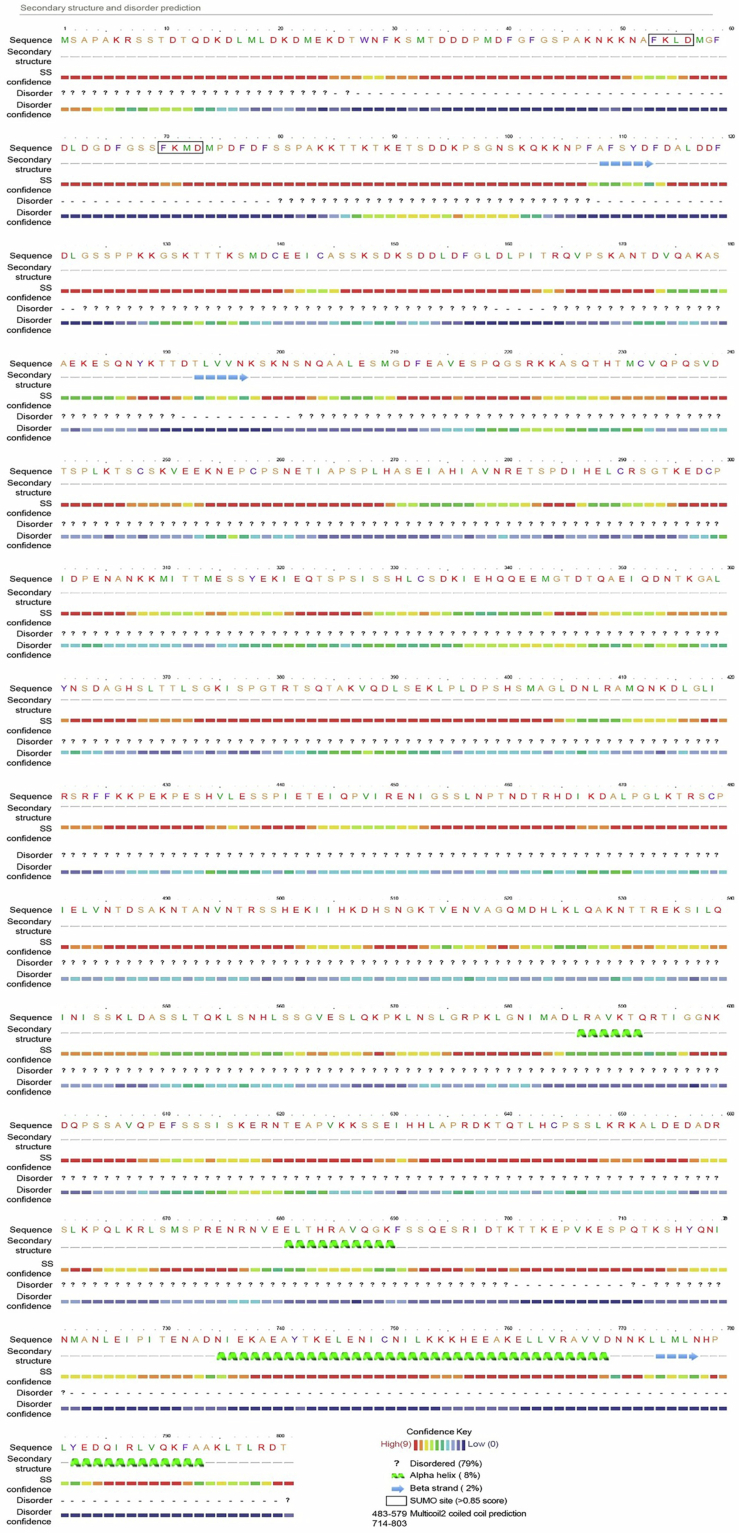
Phyre2.2 SCEP3 protein secondary structure. High probability SUMO sites predicted by SUMOPlot and coiled-coils predicted by Multicoil2.

**Extended Data Fig. 9:**
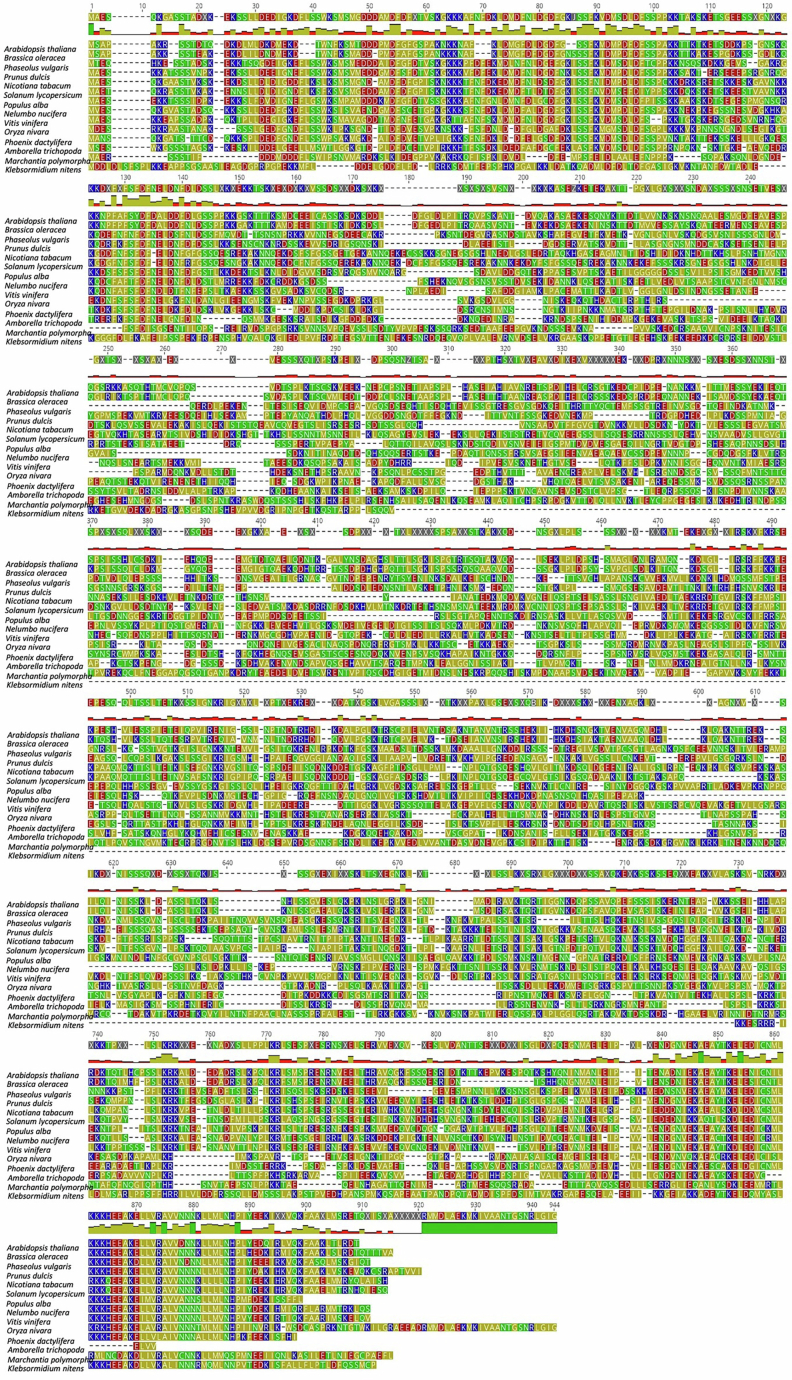
Alignment of full length SCEP3 plant homologs. The consensus sequence above the alignment is annotated with amino acid number from the N-terminal end, along with a histogram indicating level of conservation. Protein sequences were obtained from UNIPROT, Genbank and Ensembl Plants and aligned in Geneious 11.1.5 using the Geneious Alignment plugin with default settings.

The *SCEP3* gene is predicted to express seven differentially spliced transcripts according to The *Arabidopsis* Information Resource, but only variant 5 was detected in our samples, and it contained an additional 18 bp in the 3′ UTR (GenBank PP962290). Splice variant 5 encodes an 801-amino-acid protein predicted to be 79% intrinsically disordered with a compositional bias of polar and charged residues and three α-helices at the C terminus (Extended Data Fig. 8). In the plant kingdom, SCEP3 is conserved in only the first ~150 residues of the N terminus and the last ~80 residues of the C terminus (Fig. 4a and Extended Data Fig. 9). A conserved FAFSYDF site (Fig. 4b) present in the N terminus is predicted to form a β-strand downstream of two high-probability SUMO sites (FKLD and FKMD), and at the C terminus the last ~80 residues are predicted to form two coiled-coils (Extended Data Figs. 8 and 9). SYP-4 (*C. elegans*) and SIX6OS1 (mammals) are predominantly intrinsically disordered SC proteins with predicted coiled-coils and phenylalanine (FxF) repeats^48,49,51^. However, in SYP-4 and SIX6OS1, the FxF repeats are located at the C termini and the coiled-coils in the N termini, inverse to SCEP3 (Fig. 4b).

### SCEP3 interacts with itself and SC proteins

**Fig. 5:**
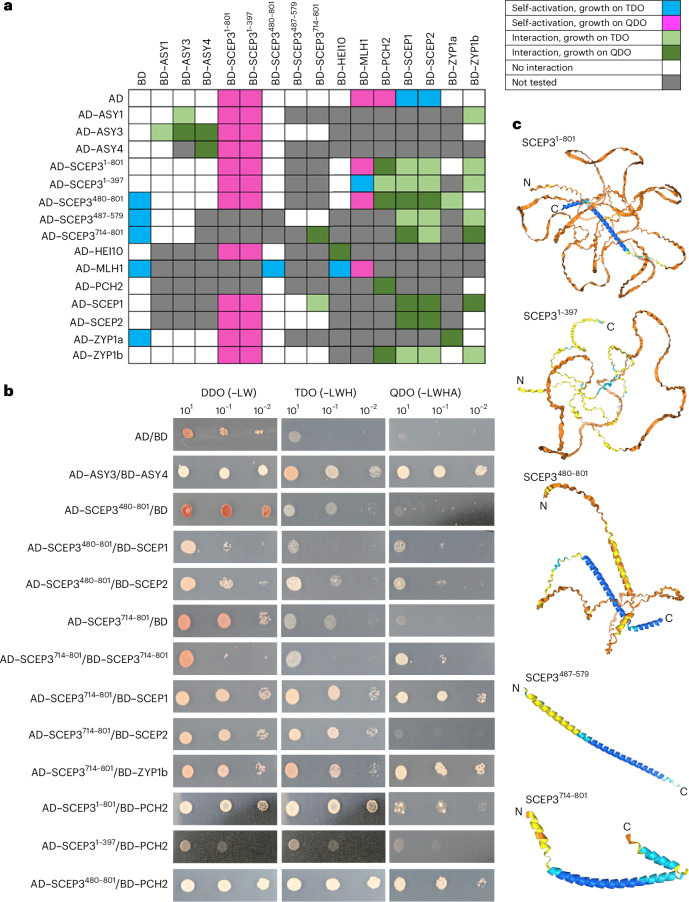
SCEP3 strongly interacts with SC proteins in Y2H. **a**, A summary table of proteins tested for interactions. **b**, Double dropout (DDO (−LW), lacking leucine and tryptophan), triple dropout (TDO (−LWH), lacking leucine, tryptophan and histidine) and quadruple dropout (QDO (−LWHA), lacking leucine, tryptophan, histidine and adenine) plates showing the growth of yeast with controls and tested gene combinations spotted in serial dilutions. AD, activation domain; BD, binding domain. **c**, AlphaFold 3 predictions of SCEP3 protein structures of the sections used in Y2H.

SCEP3 yeast-two-hybrid (Y2H) analysis was performed with axis proteins (ASY1, ASY3 and ASY4), SC proteins (ZYP1a, ZYP1b, SCEP1 and SCEP2), SC-associated protein PCH2 and class I CO proteins (HEI10 and MLH1) (Fig. 5a,b and Supplementary Information). Protein interactions that had previously been reported (AD–ASY3/BD–ASY3, AD–ASY3/BD–ASY4, AD–ASY4/BD–ASY4, AD–SCEP1/BD–SCEP1, AD–SCEP2/BD–SCEP2 and AD–SCEP1/BD–SCEP2) were repeated as positive controls, and these grew on high-stringency quadruple dropout (QDO) medium. No interactions were detected between ASY1/SCEP3, ASY3/SCEP3 or ASY4/SCEP3, or between class I CO proteins HEI10/SCEP3 and MLH1/SCEP3.

The full-length BD–SCEP3 could not be tested for protein–protein interactions due to autoactivation with the empty AD-vector control. BD–SCEP3 was therefore divided into two halves containing the N terminus (residues 1–397) and the C terminus (residues 480–801), and the N terminus showed strong autoactivation (Fig. 5a). However, the full-length AD–SCEP3 did not autoactivate and was tested for interactions. The AD–SCEP3 C terminus grew on QDO with BD–SCEP1 and BD–SCEP2 and on triple dropout (TDO) with BD–ZYP1a (Fig. 5a–c). The two predicted coiled-coils were tested separately to determine specific interactions with SC proteins. The AD–SCEP3 mid-coil (residues 487–579) grew on TDO with BD–SCEP1, BD–SCEP2 and BD–ZYP1b, and the conserved AD–SCEP3 C-terminal coil (residues 714–801) grew on TDO with BD–SCEP2 and on QDO with itself, BD–SCEP1 and BD–ZYP1b (Fig. 5a–c). In addition, AD–SCEP3N grew on TDO with BD–SCEP1 and BD–ZYP1b (Fig. 5a). AD–PCH2/BD–PCH2 and AD–ZYP1b/BD–PCH2 grew on QDO as previously shown^39^. Full-length AD–SCEP3/BD–PCH2 grew on QDO, as did AD–SCEP3^480–801^/BD–PCH2, and AD–SCEP3^1–397^/BD–PCH2 grew on TDO. These data indicate that SCEP3 interacts with itself and other SC components at multiple sites.

The analysis also showed that ZYP1a strongly interacts with itself, whereas AD–ZYP1b/BD–ZYP1b grew on the less stringent TDO, but no interactions were detected between ZYP1a and ZYP1b. AD–SCEP1 grew on QDO with BD–ZYP1b, and both BD–SCEP1/AD–ZYP1b and AD–ZYP1b/BD–SCEP2 grew on TDO. In addition, AD–HEI10 and BD–HEI10 grew on QDO, indicating that HEI10 interacts with itself, but no other interactions with HEI10 were detected (Fig. 5).

**Extended Data Fig. 10:**
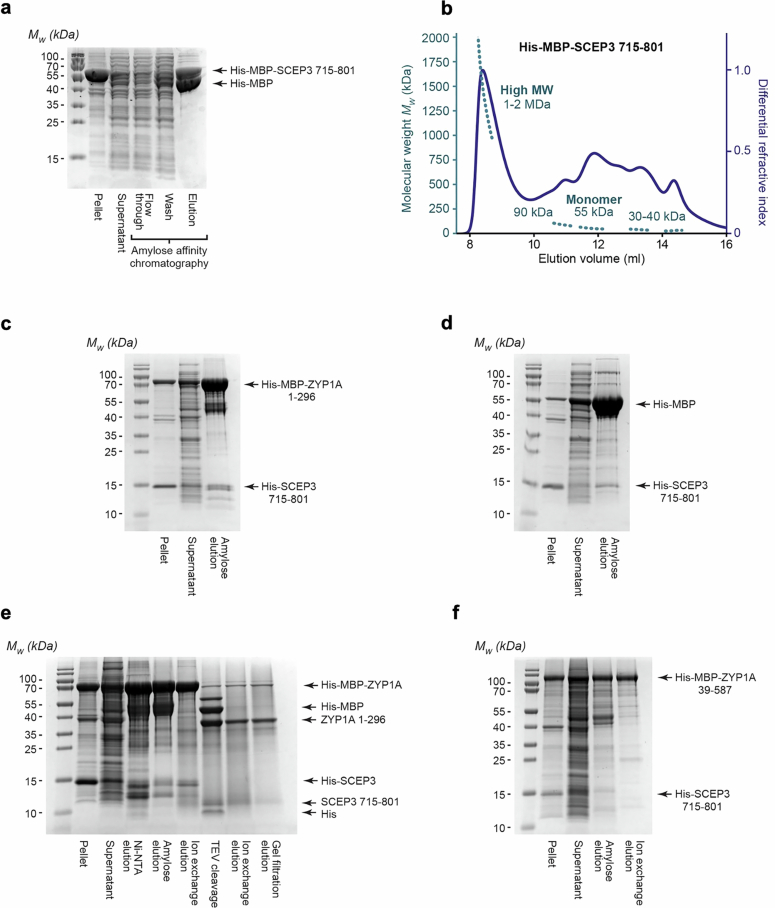
SCEP3’s C-terminal coiled-coil region does not form a stable complex with ZYP1A. **a** Expression and amylose affinity purification of His-MBP-SCEP3 715-801, showing the pellet and supernatant following recombinant expression in bacteria, alongside the amylose elution. **b** Size exclusion chromatography coupled to multi-angle light scattering (SEC-MALS) analysis in which differential refractive index (dRI; solid lines) is shown with fitted molecular weights (*Mw*; dashed lines) plotted across elution peaks. MBP-SCEP3 715-801 is largely a heterogeneous high molecular weight species of between approximately 1-2 MDa, with the presence of some monomer (55 kDa) and intermediate species (including a peak at approximately 90 kDa). The theoretical monomer mass of MBP-SCEP3 715-801 is 55 kDa. The lower molecular weight species (30-40 kDa) correspond to degraded material including free MBP. Data were collected at a protein concentration of 8 mg/ml. **c**, **d** Amylose pulldowns of His-SCEP3 715-801 following recombinant co-expression with **c** His-MBP-ZYP1A 1-296 and **d** free His-MBP, showing bacterial cell lysate pellets and supernatants, alongside amylose elutions. **e** Co-expression and attempted co-purification of His-MBP-ZYP1A 1-296 and His-SCEP3 715-801 by Ni-NTA, amylose and ion exchange chromatography, followed by TEV cleavage (to remove affinity tags), with subsequent ion exchange and size-exclusion chromatography. Bands corresponding to His-MBP-ZYP1A 1-296, His-SCEP3 715-801, ZYP1A 1-296 and SCEP3 715-801, and cleaved affinity tags His-MBP and His are indicated. **f** Amylose pulldown and subsequent ion exchange chromatography purification of His-SCEP3 715-801 following recombinant co-expression with His-MBP-ZYP1A 39-587, showing bacterial cell lysate pellets and supernatants, with amylose and ion exchange elutions.

We therefore tested key findings biochemically. Size-exclusion chromatography multi-angle light scattering (SEC-MALS) analysis of the recombinantly expressed protein revealed that SCEP3’s C-terminal coil (residues 715–801) predominantly forms high-molecular-weight species (1–2 MDa) in vitro, in agreement with a capability for self-interaction (Extended Data Fig. 10). The heterogenous nature of the observed structures suggests that SCEP3’s self-association may combine with binding to other components to form specific heterotypic coiled-coil oligomers that act as coherent building blocks of the SC. In agreement with our Y2H data, it was previously proposed that SCEP3 may bind to the N-terminal end of ZYP1 (ref. ^52^). We thus tested this via amylose pulldown following co-expression of the MBP-tagged N-terminal end of ZYP1a (residues 1–296) with SCEP3’s C-terminal coil (residues 715–801). We observed that a small amount of SCEP3 was pulled down, in a manner equivalent to the free MBP control (Extended Data Fig. 10). We tested this stringently by purifying the co-expressed proteins through multiple steps of chromatography, in which we observed almost complete dissociation by the final step of SEC (Extended Data Fig. 10). Hence, we observed no interaction other than a low level consistent with background non-specific binding. We wondered whether complex formation may require a much longer construct encompassing the majority of ZYP1’s coiled-coil. However, co-expression with MBP–ZYP1a (residues 39–587) pulled down only the same background level of SCEP3 C-terminal coil by amylose affinity, which completely dissociated upon subsequent ion exchange chromatography (Extended Data Fig. 10). We thus observed no evidence of robust heterotypic coiled-coil formation between ZYP1 and SCEP3. Instead, SCEP3’s C-terminal coil may form a heterotypic coiled-coil with multiple SC components. Despite additional co-expression experiments with other SC components, we have not been able to identify any combination that leads to coherent complex formation. Thus, this remains an important unknown for future biochemical studies, which may require the presence of additional currently unidentified plant SC components.

### Loss of *SCEP3* increases CO frequency

**Fig. 6:**
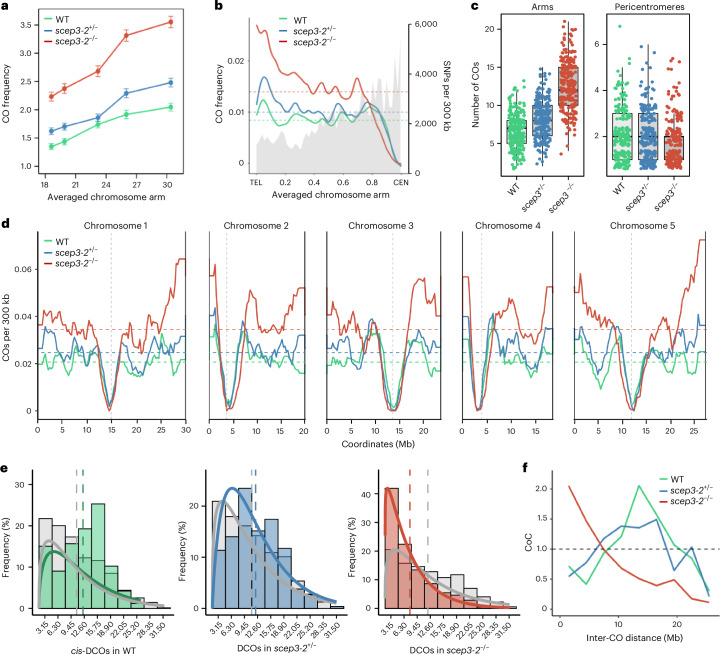
*scep3* mutants exhibit a significantly increased number of COs and pronounced CO clustering. **a**, Differences in total CO numbers per chromosome in the wild type, *scep3*^+/−^ and *scep3*^−/−^. Mean CO numbers are shown; the whiskers represent standard errors. The data are from *n* = 259 wild-type, 269 *scep3*^+/−^ and 264 *scep3*^−/−^ F_2_ individuals (2,172, 2,686 and 3,686 COs, respectively). **b**, CO frequency per F_2_ individual, averaged within 300-kb windows along a proportionally scaled chromosome arm oriented from telomere (TEL) to centromere (CEN). SNP density per 300 kb is shown in grey shading. The horizontal dashed lines indicate mean values. **c**, Number of COs within chromosome arms and pericentromeric regions and total counts in *scep3* mutants compared with the wild type. Each dot represents one F_2_ individual; the data points are jittered along both the *x* and *y* axes to improve visualization and reduce overlap. The box plots summarize the distribution per genotype, with the centre line indicating the median and the upper and lower bounds indicating the 75th and 25th percentiles, respectively. The whiskers extend to 1.5× the interquartile range beyond the quartiles. Pericentromeres are defined as regions flanking centromeres that have higher-than-average DNA methylation. Statistical significance was assessed using two-sided Wilcoxon tests with Bonferroni correction. The data are from the same set of F_2_ individuals as in **a**. **d**, Similar to **b** but showing CO frequency plotted across all five *Arabidopsis* chromosomes, averaged in 300-kb windows. The vertical dashed lines mark centromere positions. **e**, Histograms showing the distribution of inter-CO distances for observed data (coloured bars) versus randomly simulated distances (grey bars) in the wild type (*n* = 166), *scep3*^+/−^ (*n* = 264) and *scep3*^−/−^ (*n* = 625). Gamma-fitted curves are overlaid for both observed (coloured lines) and expected (grey lines) distributions. **f**, CoC analysis. The *y* axis shows the CoC, while the *x* axis indicates the distance between intervals (in Mb). A CoC value of 1 reflects independent CO formation, values below 1 suggest interference and values above 1 at short distances indicate CO clustering.

To investigate the genome-wide distribution of COs, we employed a sequencing-based approach with F_2_ populations derived from Col/Landsberg *erecta* (L*er*) hybrids carrying mutations in *scep3*. *scep3-2*^+/−^ (Col) was crossed with *scep3-4*^+/−^ (L*er*)^52^, and the resulting plants were genotyped and allowed to self-pollinate. Seeds collected from *scep3-2* *scep3-4* (hereafter *scep3*^−/−^), *scep3-2* *SCEP3* (hereafter *scep3*^+/−^) and *SCEP3* *SCEP3* (wild-type) plants produced F_2_ populations that were sequenced to identify Col/L*er* genotype switches corresponding to CO sites. In total, we analysed 259 wild-type, 269 *scep3*^+/−^ and 264 *scep3*^−/−^ individuals, identifying 2,172, 2,686 and 3,686 COs, respectively. This analysis revealed that COs are significantly more numerous in *scep3* mutants than in the wild type, especially towards the chromosome ends (Fig. 6a,b). The *scep3*^−/−^ mutant exhibits an average of 14 COs per individual, which is significantly higher than the 8.39 COs per individual observed in the wild type (Fig. 6c; *P* = 3.43 × 10^−72^; Dunn Kruskal–Wallis multiple comparisons with Bonferroni correction). *scep3*^+/−^ exhibits an average of 9.99 COs per individual, which is significantly higher than the wild type (*P* = 3.09 × 10^−9^) and indicates haplo-insufficiency of *SCEP3*.

Analysis of CO distribution along an averaged chromosome arm revealed an increase in CO frequency in the *scep3*^−/−^ mutant primarily in interstitial and sub-telomeric regions, whereas pericentromeric regions exhibited a reduction in recombination (Fig. 6c). To further characterize this phenomenon, we quantified CO counts separately for chromosome arms and pericentromeric regions. Pericentromeres are defined as the contiguous regions flanking the centromeres characterized by higher DNA methylation than the genome-wide average, while chromosome arms encompass the remainder of the genome^53^. This analysis confirmed a statistically significant decrease in CO numbers in pericentromeric regions in the *scep3*^−/−^ mutant compared with the wild type and *scep3*^+/−^ (Fig. 6c; *P* < 3.1 × 10^−2^; Dunn Kruskal–Wallis multiple comparisons with Bonferroni correction). Notably, *scep3*^+/−^ did not show a change in pericentromeric CO frequency relative to the wild type. Comparison of CO numbers per chromosome demonstrated that increases in both *scep3*^+/−^ and *scep3*^−/−^ are most pronounced on the two longest chromosomes (Fig. 6d).

### Strong negative interference is observed in *scep3*

Although our analysis of COs was based on sequencing F_2_ individuals (which result from two independent meioses), by filtering for the parental–heterozygous–parental genotype pattern (for example, Col/Col–Col/L*er*–Col/Col and L*er*/L*er*–Col/L*er*–L*er*/L*er*), it is possible to identify *cis*-double COs (*cis*-DCOs) and measure their distances, providing insights into CO interference^54–56^. We therefore analysed the distribution of *cis*-DCO distances and compared them to the expected distribution of distances between two randomly selected CO sites. In the wild type, most *cis*-DCOs occur at distances between approximately 9 and 18 Mb (Fig. 6e). A similar distribution of *cis*-DCO frequencies was observed in *scep3*^+/−^, although this distribution is shifted towards shorter distances with a peak of ~9.45 Mb (Fig. 6e). In contrast, *scep3*^−/−^ exhibits a drastic shift of *cis*-DCOs towards very short distances where the frequency in the shortest distance bin of the histogram is twice as high as expected under a random distribution model. Subsequently, we performed a coefficient of coincidence (CoC) analysis, which confirmed these observations (Fig. 6f). On the basis of these results, we conclude that while *scep3*^+/−^ shows only a mild reduction in CO interference compared with the wild type, *scep3*^−/−^ is characterized by strong negative interference and clustering of COs.

## Discussion

### SCEP3 links meiotic recombination with chromosome pairing

In the wild type, ~187 SCEP3N foci per cell localize to the chromosome axes at leptotene, of which ~84% are dependent on DSB formation by Spo11. In the absence of DMC1, ~232 SCEP3N foci were observed, consistent with an increase in DSB numbers due to a lack of homologue engagement and a feedback mechanism that maintains the chromosomes in a DSB competent state^57,58^. In the *scep3* mutant, chiasmata were significantly reduced in all chromosomes except for chromosome 4, which contains the nuclear organizing region, although it is worth noting that multiple COs are likely to be concealed within a single chiasma by negative interference. In the *Arabidopsis* *hop2* mutant, the nuclear-organizing-region-containing short arms of chromosomes 2 and 4 paired against a nucleus-wide lack of pairing^59^, consistent with SCEP3 being less required for promoting the pairing of chromosome 4, but necessary for chromosomes 1, 2, 3 and 5. The shift in CO distribution from pericentromeric regions towards the chromosome ends in the mapping data is also consistent with reduced pairing as shown in *asy1* and *asy4* mutants^33,36^. In the *scep3* *asy1* double mutant, chiasmata were significantly reduced when compared with the *asy1* single mutant, indicating that SCEP3 has a pro-CO SC-independent role. The pro-CO role may be achieved through the formation of SCEP3 interaxial bridges that are observed in the wild type. In addition, SCEP3 may have an SC-independent anti-CO role, as COs rarely cluster in the *zyp1* mutant, but CO clusters are significantly more frequent in the *scep3* mutant^38^. The SCEP3C antibody has a similar pattern of localization to SCEP3N during zygotene and pachytene when the axes have been remodelled by PCH2 and ZYP1 into lateral elements^40^. At leptotene, SCEP3N foci numbers are significantly greater than SCEP3C in the wild type, but in the *asy1* mutant both are localized as large foci that may reflect masking of the SCEP3C antigen by ASY1.

### SCEP3 is required for SC initiation

A small proportion of the SCEP3 axis-associated foci enlarge and initiate the SC. As the SC polymerizes, SCEP3 foci translocate from the axes to the SC central region and transition into larger foci. In the absence of SCEP3, chromosome axes align at 419 nm but are not juxtaposed to 263 nm as in the wild type. ZYP1N and ZYP1C colocalize to *scep3* meiotic chromosomes as 22 foci, which may represent SICs, consistent with previous estimates of 20–25 in *Arabidopsis*^60^. In the *scep3* heterozygous mutant, ZYP1 completely polymerizes, but large stretches contain very little or no SCEP3 protein. This may indicate that ZYP1 polymerization can proceed with very little SCEP3 protein or that SCEP3 was initially present in these regions but was degraded or translocated to other regions of the SC as proposed in *C. elegans*^61^. In the *scep3* *zyp1* mutant, chiasmata were significantly reduced when compared with *zyp1* but not when compared with *scep3*, suggesting that SCEP3 acts upstream of ZYP1. In the *zyp1* mutant, SCEP3 foci do not extend, indicating interdependency of both proteins for SC formation.

### SCEP3 is structurally similar to SC proteins across kingdoms

The immunolocalization experiments revealed that SCEP3 forms foci that may consist of protein aggregates/condensates that increase in size as prophase I progresses, as well as forming bridges between physically separate axial regions. The SCEP3 N-terminal FxF and C-terminal coiled-coil are conserved across plant species, whereas the unconserved internal region is predominantly composed of polar and charged residues that may act as a linker or provide a sticky interface for proteins to bind via electrostatic interactions^62^. The N-terminal FxFs of SCEP3 are predicted to form a hydrophobic β-strand adjacent to two high-probability SUMO sites. The SCEP3 N terminus shares similarity to the C-terminal FxFs of SC proteins SYP-4 in *C. elegans*^48^ and SIX6OS1 in mammals^49^. Weak hydrophobic interactions are required for SC formation in *C. elegans*^61^, but SYP-4’s FxFs are not necessary for synapsis^48^. However, they are crucial for CO assurance and CO interference by recruiting ZHP-3, a predicted E3 ligase homologous to HEI10 (ref. ^48^). Neither the SCEP3 full-length protein nor the FxF-containing N terminus interacted with HEI10 in Y2H, whereas HEI10 interacted with itself, although modification of the two high-probability N-terminal SUMO sites may be required for an interaction in planta. The SCEP3 C-terminal coiled-coil grew on the most stringent QDO medium with itself, SCEP1, SCEP2 and PCH2 and on TDO with ZYP1a. These interactions were tested biochemically with pull-down assays, but only the SCEP3 C-terminal coiled-coil strongly associated with itself in large 1–2-MDa complexes, suggesting that it may be responsible for forming the large cytological signals that are observed during prophase I, but no strong interaction was identified with ZYP1 as proposed by Feng et al.^52^. In Y2H no interactions were detected between SCEP3 and axis proteins ASY1, ASY3 or ASY4 or class I CO protein MLH1. SCEP3 may also be the functional homologue of Corolla in fruit fly and ECM11 in budding yeast, as both SC central element proteins possess coiled-coils with intrinsically disordered regions^63^.

### SCEP3 prevents strong negative CO interference

In SC mutants *zyp1* (ref. ^37^), *scep1*, *scep2* (ref. ^35^) and *scep3* (ref. ^52^), positive CO interference was abolished, and recombination increased towards the chromosome ends. Through pollen fluorescent-tagged line (FTL) analysis, we previously showed that recombination in *zyp1* increased ~1.5-fold with the loss of CO interference (interference ratio of 0.92 in I3)^38^. The recombination data generated in this current study reveal that CO frequency in *scep3*^−/−^ increases to levels previously reported for SC mutants (~14 COs per meiosis) as well as haplo-insufficiency of *SCEP3*. However, unlike the analysis of *scep3* in ref. ^52^, we observed strong negative CO interference via CO mapping and HEI10 clustering in both the asynaptic *scep3*^−/−^ and synaptic *scep3*^+/−^ mutants. The analysis in ref. ^52^ was restricted to chromosomes with exactly two COs, which has underestimated clustering, whereas our approach accommodates higher CO numbers and consistently reveals strong negative interference at short intervals. COs in *scep3* are not randomly distributed but form preferentially towards chromosome ends and cluster, similar to *asy4* (ref. ^33^) and *atm*^64^ mutants. A weaker clustering effect is also observed in *scep1* (ref. ^35^) and *zyp1* *HEI10-OE*^65^, probably reflecting increased loading of the pro-CO factor HEI10 at chromosome ends. In the absence of interference, this promotes local CO clustering, consistent with elevated short *cis*-DCOs in distal regions of *scep3*^−/−^.

### SCEP3 maintains the obligate CO

In the absence of SCEP3, a prevalence of univalents indicates that CO assurance is abolished. The class II CO pathway does not appear to be affected in *scep3* as chiasma counts were not significantly different between *msh5* *scep3*^−/−^, *msh5* *scep3*^+/−^ and *msh5*, so it may be that SCEP3 only mediates CO patterning of the class I CO pathway. In addition, MSH5-dependent chromatin bridges were observed in the *scep3* mutants between non-homologous chromosomes, homologous chromosomes and sister chromatids, which may represent unresolved class I CO recombination intermediates or telomeric connections^50^. This phenotype contrasts with the *zyp1* mutant, where class II COs were significantly reduced and univalent pairs were about ninefold less frequent^38^. In plants, class II COs appear to reflect a proportion of designated class I COs^66^. It would therefore follow that class I COs have been designated prior to or at the same time as synapsis initiation in the *scep3* mutant, consistent with budding yeast^67^.

### The role of SCEP3 in CO patterning

**Fig. 7:**
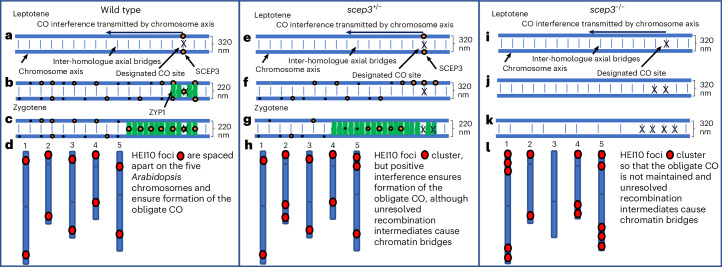
SCEP3 CO model. **a**, In the wild type, chromosome pairing through axial bridges and DMC1-mediated strand invasion promote correct homologue alignment, and a small number of sites are designated for CO formation. The designated site could transmit an interference signal via the axis as predicted by the beam-film model^68^. SCEP3 foci localize to the axis at the designated CO site and stabilize it. **b**, SCEP3 protein promotes the installation of the SC and transitions from the axis to the central region. The designated CO site may stabilize adjacent axial bridges, so SCEP3 and ZYP1 remove these by recruiting PCH2 to the central region, thereby preventing negative interference. **c**, ZYP1 polymerizes, enabling SCEP3 and PCH2 to remove axial bridges at a distance from the designated CO site, thereby preventing undesignated CO formation and imposing positive CO interference. **d**, Class I COs are spaced apart as shown by HEI10 localization, and each chromosome pair receives at least one CO. **e**, In *scep3*^+/−^, the same process as in **a** occurs except the dynamics may be altered due to a reduction in expression of *SCEP3*. **f**, The designated CO may stabilize adjacent sites, but reduced SCEP3 expression may be insufficient to prevent these sites from maturing into COs. **g**, ZYP1 polymerizes, but reduced SCEP3 protein in the SC central region may not prevent undesignated sites at a distance to the designated site from maturing into a CO. **h**, At late prophase, HEI10 foci cluster on the SC and increase in number compared with the wild type. **i**, In *scep3*^−/−^, a designated CO may or may not mature in the absence of SCEP3. **j**, A designated CO may stabilize an adjacent site to mature into a CO. **k**, ZYP1 does not polymerize, but without the pro-CO role of SCEP3, some interaxial interactions may not be stabilized, thereby losing the maturation of designated CO sites. **l**, HEI10 clusters, but multiple sites sequester it, which leads to the loss of CO assurance.

These data indicate that SCEP3 performs three major roles in implementing CO assurance: (1) it promotes CO formation independent of the SC, (2) it prevents clustering of COs independent of the SC and (3) it prevents extra COs from forming dependent on the SC (Fig. 7). During leptotene, SCEP3 forms numerous DSB-dependent axis-associated foci. The majority are small, but a subset emerge as larger foci that colocalize with ZYP1 and represent SICs. We hypothesize that the larger SCEP3 foci form at designated CO sites and stabilize axial bridges. The clustered SCEP3 foci then prevent adjacent DSB sites from maturing into COs, thereby preventing negative CO interference. If negative interference is not prevented, HEI10 accumulation at these sites sequesters the protein away from other chromosomes, leading to the loss of CO assurance (Fig. 7). In *Arabidopsis* 20–25 SICs emerge at late leptotene/early zygotene^60^, consistent with our current estimate of 22. In common with other species, these sites are likely to exhibit interference, of which ~40% will be coincident with class I COs^67^. This suggests that the initial stages of CO patterning occur prior to SC extension, consistent with the beam-film model, which is posited to explain CO patterning in budding yeast^68^. A mechanical model that integrates chromosome pairing, synapsis initiation and CO patterning may explain the striking similarity between CO clustering in *scep3* and the *pss1* mutant, which lacks rapid chromosome movement during prophase I^69^. However, it has recently been proposed that CO patterning arises due to HEI10 coarsening^41,42^. On the basis of our observations, we suggest that HEI10 coarsening may act to select ~9 CO sites from among the 20–25 SICs. It seems feasible that the initiation of HEI10 coarsening would be favoured at early SICs and that coalescence with later SICs would be SC-mediated via SCEP3’s FxFs. This would suggest that patterning of class I COs occurs as a stepwise process commencing at late leptotene/early zygotene and finishing at pachytene.

## Methods

### Plant materials

*scep3-2* (SALK_098044), *scep3-4* (ref. ^52^), *scep3-5* (SALK_023936), *scep3-6* (GK500E03), *asy1-4* (SALK_046272)^40^, *asy3-1* (SALK_143676)^32^, *asy4-3* (ref. ^70^), *pch2-1* (SAIL_1187_C06)^40^, *msh5-1* (SALK_110240)^71^, *dmc1* (SAIL_170_F08)^33^, *spo11-1-5* (SALK_131704) and *hei10-2* (SALK_014624)^19^ were acquired from the Nottingham Arabidopsis Stock Centre (https://arabidopsis.info/), and z*yp1a-2* was previously generated^38^.

### Fertility analysis

Twenty dried siliques from the fourth to tenth nodes of primary, secondary, tertiary and quaternary stems were isolated and counted for the wild type and *scep3* mutants. Alexander-stained pollen was assessed for viability and counted^72^.

### Cloning and sequencing of *SCEP3*

Total RNA was extracted from *A. thaliana* (Col-0) floral buds as described in ref. ^44^ and DNased using a Turbo DNA-free kit, and cDNA was synthesized with RevertAid (www.thermofisher.com/). The 3′ end of *SCEP3* was amplified via RACE PCR using an anchored oligo(dT) primer^73^ followed by two rounds of PCR with the anchor primer and the primers SCEP3-3PR-F1 (first PCR) and SCEP3-3PR-F2 (second PCR) in conjunction with Q5 High Fidelity Master Mix (www.neb.com). Purified PCR products were ligated into pDrive (www.qiagen.com/) and sequenced (https://eurofinsgenomics.eu/). All primers used in this study are shown in Supplementary Table 5.

### Antibody production

On the basis of our cloned and sequenced full-length *SCEP3* transcript, peptides were designed for the SCEP3 N-terminal amino acids 61–75 (C+DLDGDFGSSFKMDMP) and for the SCEP3 C-terminal amino acids 689–703 (C+GKFSSQESRIDTKTT) and synthesized by Eurogentec (www.eurogentec.com/en/). The speedy 28-day programme was used to generate SCEP3N (rat) and SCEP3C (rabbit) polyclonal antibodies.

### Cytological procedures

*Arabidopsis* inflorescences were fixed in 3:1 ethanol:acetic acid and stored at −20 °C. Slides were prepared from these buds, and fluorescence in situ hybridization was performed as described previously^2^. HEI10 foci counts were performed first by subjecting the slides to the microwave technique^74^ and then incubating with rabbit anti-HvHEI10 (ref. ^66^) and rat anti-SMC3 (1:200)^32^, followed by anti-rabbit Dylight 594 (https://vectorlabs.com/) and anti-rat Alexa Fluor 488 (www.thermofisher.com/) secondary antibodies. A Nikon NI-E fluorescence microscope was used to capture meiotic nuclei, and the thresholding software tool NIS-elements (www.microscope.healthcare.nikon.com/) was employed to count the HEI10 foci on the basis of size with a lower threshold cut-off of 0.001 µm^2^. Super-resolution microscopy was performed with the Zeiss LSM 980 Airyscan 2 in the Advanced Imaging Facility at the University of Leicester using default Airyscan processing. SCEP3N foci were captured with the Zeiss LSM 980 Airyscan 2, and automatic intensity thresholding using the default settings was performed in ImageJ (Fiji 2.9.0/1.54p) at 0.001 µm^2^. This thresholding step robustly excludes background signal and ensures that the apparent foci sizes signify real fluorescence signals above background. Axis width measurements and analyses were performed^38^. Slides were prepared for immunolocalization of fresh material as described previously^2^, and proteins were stained using the following primary antibodies at a concentration of 1:500: rat anti-AtZYP1-C^75^, rat/guinea pig anti-AtZYP1-C^34^, rabbit anti-AtZYP1-N^71^, guinea pig/rat anti-ASY1 (ref. ^76^), rabbit anti-ASY3 (ref. ^32^), rabbit anti-PCH2 (ref. ^40^) and rabbit anti-REC8 (ref. ^77^).

### Genome-wide CO mapping

To generate genomic CO genotyping-by-sequencing landscapes for Col × L*er* crosses in the genetic background of the wild type, *scep3-2*^Col^*/SCEP3*^L*er*^ and *scep3-2*^Col^*/scep3-4*^L*er*^, heterozygous *scep3-2*^Col^ plants were crossed with heterozygous *scep3-4*^L*er*^. Genomic DNA was extracted from F_2_ individuals derived from three independent plants per genotype. DNA isolation was performed using the cetyltrimethylammonium bromide method, following the protocol described in ref. ^78^. DNA quality was assessed via electrophoresis on a 1% agarose gel. For tagmentation, 1 µl of DNA (5 ng µl^−1^) was combined with 1 µl of Tagmentation Buffer (40 mM Tris-HCl, pH 7.5; 40 mM MgCl_2_), 0.5 µl of dimethylformamide, 2.35 µl of nuclease-free water and 0.05 µl of in-house Tn5 tagmentase pre-loaded with annealed linker oligonucleotides, as previously described^79^. The tagmentation reaction was carried out at 55 °C for 2 min and terminated by the addition of 1 µl of 0.1% SDS, followed by incubation at 65 °C for 10 min. Tagmented DNA was amplified using the KAPA2G Robust PCR kit (www.sigmaaldrich.com/) with custom P5 and P7 indexing primers, assigning each sample a unique combination^80^. The quality of the amplified libraries was verified on a 2% agarose gel. Successfully amplified libraries were pooled and subjected to size selection, where DNA fragments between 500 and 700 bp were excised from a 2% agarose gel and purified using a Gel Extraction Kit (www.aabiot.com/). Final library concentration was assessed using a Qubit 2.0 fluorometer. Paired-end sequencing was performed on an Illumina NovaSeq X 10B platform. Demultiplexed reads were aligned to the TAIR10 Col-0 genome reference sequence with BowTie2 (ref. ^81^). The resultant BAM files were sorted, and single nucleotide polymorphisms (SNPs) were called with SAMtools and BCFtools^82^, on the basis of a comprehensive list of polymorphisms from a large-scale Col × L*er* F_2_ population^78^. The resulting tables of SNPs were filtered to retain only those exhibiting high mapping quality (>100) and high coverage (>2.5×). Libraries with <75,000 reads associated with SNPs were excluded from the analysis. CO calling used the TIGER pipeline on the filtered files^83^. CO distribution frequencies were binned into scaled windows and cumulatively aggregated across chromosome arms. The raw FASTQ data can be found in the NCBI Sequence Read Archive under the BioProject accession code PRJNA1285115.

### *cis*-DCO and CoC analysis

*cis*-DCO distances were defined as the physical distances between transitions of parental–heterozygous–parental genotypes (that is, Col/Col–Col/L*er*–Col/Col or L*er*/L*er*–Col/L*er*–L*er*/L*er*) calculated from genotyping-by-sequencing (GBS) data. The distribution of observed distances was then compared to the expected distribution under the assumption of random CO placement. To generate the expected distribution, 200 pairs of CO midpoints were randomly sampled from the total set of identified COs for each genotype, analysed separately for each chromosome. Frequencies of observed and expected *cis*-DCO distances were calculated and visualized in 3.15-Mb bins, including the median values for both distributions. Short *cis*-double COs with a distance <5 Mb between the two COs were also analysed. For each event, the midpoint was calculated, and the distance to the nearest chromosome end was normalized by chromosome length. To assess whether short *cis*-DCOs in the *scep3* mutant were non-randomly positioned, a permutation test was performed: for each observed *cis*-DCO, 10,000 random positions were generated on the same chromosome, the normalized distances were calculated and the mean of each permutation was compared to the observed mean. Empirical *P* values were obtained as the proportion of permutations with mean distances at least as extreme as the observed. To assess differences between observed and expected distributions, gamma distributions were fitted to each dataset using the fitdist function from the fitdistrplus R package^84^. For CoC analysis, expected *cis*-DCO distances were scaled according to the observed CO frequency for each genotype^85^. Finally, CoC values were calculated by dividing the observed *cis*-DCO frequency by the expected frequency in each interval.

### Protein structure and sequence analysis

SCEP3 intrinsically disordered regions, α-helices and β-strands were predicted by Phyre2.2 (https://www.sbg.bio.ic.ac.uk/phyre2/html/page.cgi?id=index)^86^, coiled-coils were predicted by Multicoil2 (https://cb.csail.mit.edu/cb/multicoil2/cgi-bin/multicoil2.cgi)^87^ and SCEP3 protein structures were predicted by AlphaFold 3 (https://alphafoldserver.com/)^88^. All protein sequences were obtained from UNIPROT (https://www.uniprot.org/) (apart from XP_019078001 retrieved from GenBank) and aligned in Geneious v.11.1.5 using the alignment plugin with the default settings. The accession numbers were as follows: A0A1P8B5T3 (*A. thaliana*), A0A0D3DEV4 (*Brassica oleracea*), V7C8M9 (*Phaseolus vulgaris*), A0A5E4G8G5 (*Prunus dulcis*), A0A1S4DGP8 (*Nicotiana tabacum*), A0A3Q7FQ30 (*Solanum lycopersicum*), A0A4U5QIQ3 (*Populus alba*), A0A1U8BCW5 (*Nelumbo nucifera*), XP_019078001 (*Vitis vinifera*), A0A0E0FTB0 (*Oryza nivara*), A0A8B9ADZ6 (*Phoenix dactylifera*), W1Q102 (*Amborella trichopoda*), A0A2R6XGW7 (*Marchantia polymorpha*), A0A1Y1HSJ6 (*Klebsormidium nitens*), Q9N5K3 (SYP-4, *C. elegans*) and Q9CTN5 (SIX6OS1, *Mus musculus*).

### Y2H analysis

*ASY1*, *ASY3* and *ASY4* Y2H constructs from ref. ^27^ were used. Full-length coding regions of *ZYP1a* and *ZYP1b* were amplified using Q5 DNA polymerase (www.neb.com) with the primers ZYP1F1 and ZYP1R1, cloned into pDrive (www.qiagen.com/) and sequenced by eurofinsgenomics.eu/. NcoI and BamHI were used to digest and ligate *ZYP1* into pGADT7 and pGBKT7 (www.takarabio.com/). The full-length coding region of *SCEP3* was cloned into pGBKT7 and pGADT7 using SCEP3F1NdeI with either SCEP3R1SalI for pGBKT7 or SCEP3R1ClaI for pGADT7. The N terminus was obtained by digesting the full-length *SCEP3* with BamHI, while the C terminus of *SCEP3* was amplified with the primers C-Terminus-F and C-Terminus-R and cloned into pGADT7 and pGBKT7 (www.takarabio.com/). *SCEP1* was amplified with the primers GBK_SCEP1_f, GBK_SCEP1_r, GAD_SCEP1_f and GAD_SCEP1_r. *SCEP2* was amplified with the primers GAD_SCEP2_f, GAD_SCEP2_r, GBK_SCEP2_f and GBK_SCEP2_r. *MLH1* was amplified with the primers MLH1F and MLH1R and cloned into EcoRI/BamHI sites of pGADT7 and pGBKT7. *HEI10* was amplified with the primers HEI10F and HEI10R and cloned into NdeI/BamHI sites of pGADT7 and pGBKT7. Y2H constructs were obtained for *PCH2* (ref. ^39^), *MRE11*, *RAD50* and *NBS1* (ref. ^15^). The pGADT7 and pGBKT7 vectors were transformed into Matchmaker Yeast Two-Hybrid Gold cells using an adapted lithium acetate protocol^89,90^, and the assay was conducted as instructed by the Clontech Matchmaker Gold system (www.takarabio.com/). Yeast cells were co-transformed and selected using a synthetic double dropout medium (−Leu/−Trp). The cells were grown for three to four days at 30 °C until visible colonies appeared. −His/−Leu/−Trp (TDO) medium was used to screen for weak protein–protein interactions, and −His/−Ade/−Leu/−Trp (QDO) medium was used to screen for strong protein–protein interactions. The liquid culture was serially diluted 1/10 and 1/100 using sterile 0.9% NaCl (w/v) supplemented with ampicillin (100 mg ml^−1^) and kanamycin (50 mg ml^−1^), and 3 µl of each dilution was plated sequentially on double dropout, TDO and QDO agar plates. The agar plates were incubated for three to five days at 30 °C and photographed with a Canon EOS 1000D digital camera.

### Recombinant protein expression and purification

Sequences corresponding to regions of *A. thaliana* ZYP1A (1–296 and 39–587) and SCEP3 (715–801) were cloned into pMAT11 vectors^91^ for expression with N-terminal His_6_–MBP tags. SCEP3 (715–801) was also cloned into a pRSF-Duet1 (www.sigmaaldrich.com/) expression vector for co-expression as a TEV-cleavable N-terminal His_6_-fusion protein with the aforementioned pMAT11 vectors. The constructs were expressed or co-expressed in BL21 (DE3) cells (www.sigmaaldrich.com/), in 2xYT media, induced with 0.5 mM IPTG for 16 h at 25 °C. Cells were lysed in 20 mM Tris pH 8.0, 500 mM KCl (and 2 mM DTT for amylose pulldowns) at 4 °C using a CF1 Cell Disruptor (https://constantsystems.com/), and lysates were clarified via centrifugation at 45,000 *g*, 4 °C for 30 min (www.mybeckman.uk/). Amylose pulldowns were performed by applying supernatants to amylose resin (www.neb.com), washing with ten column volumes of lysis buffer and eluting in 20 mM Tris pH 8.0, 500 mM KCl, 2 mM DTT and 30 mM D-maltose. For co-expressions of His_6_–MBP–ZYP1A 39–587 and His_6_–SCEP3 715–801, amylose elutions were subsequently purified via HiTrap Q HP (www.cytivalifesciences.com/) ion exchange chromatography. Co-expressions of His_6_–MBP–ZYP1A 1–296 and His_6_–SCEP3 715–801 were additionally purified via initial Ni-NTA (https://www.qiagen.com/) chromatography, followed by amylose (www.neb.com) affinity and HiTrap Q HP (www.cytivalifesciences.com/) ion exchange chromatography. Affinity tags were removed via incubation with TEV protease, and cleaved samples were purified via HiTrap Q HP ion exchange chromatography and SEC (HiLoad 16/600 Superdex 200, www.gehealthcare.co.uk/) in 20 mM Tris pH 8.0, 150 mM KCl and 2 mM DTT. Protein samples were concentrated using Amicon Ultra-4 (www.sigmaaldrich.com/) centrifugal devices and were stored at −80 °C following flash-freezing in liquid nitrogen. Protein samples were analysed via SDS–PAGE with Coomassie staining, and concentrations were determined via UV spectroscopy using a Cary 60 UV spectrophotometer (www.agilent.com/) with extinction coefficients and molecular weights calculated by ProtParam (http://web.expasy.org/protparam/).

### SEC-MALS analysis

His_6_–MBP–SCEP3 715–801 was analysed via SEC-MALS at a protein concentration of 8 mg ml^−1^ in 20 mM Tris pH 8.0, 150 mM KCl and 2 mM DTT. Samples were loaded at 0.5 ml min^−1^ onto a Superdex 200 Increase 10/300 GL (www.gehealthcare.co.uk/) column with an ÄKTA Pure (www.cytivalifesciences.com/). The column outflow was fed into a DAWN HELEOS II MALS detector (www.wyatt.com/) and then an Optilab T-rEX differential refractometer (www.wyatt.com/). ASTRA 6 software (www.wyatt.com/) was used to collect and analyse the SEC-MALS data, using Zimm plot extrapolation with a 0.185 ml g^−1^ dn/dc value to determine molecular weights from eluted protein peaks.

### Quantitative PCR analysis

Flower buds harvested from ten individual *Arabidopsis* plants were pooled to comprise each biological replicate. Three replicates were collected from each genotype (wild type, *scep3-2*^−/−^ and *scep3-2*^+/−^) and frozen in liquid nitrogen before being stored at −80 °C. Total RNA was extracted from each replicate, and genomic DNA was removed on column using an ISOLATE II RNA Plant Kit (www.meridianbioscience.com/). The RNA was further DNased with a TURBO DNA-free kit (www.thermofisher.com/) to remove any remaining genomic DNA. Concentration and purity of total RNA were determined using a Nanodrop spectrophotometer (www.thermofisher.com/). First-strand cDNA was reverse-transcribed from 1 μg of total RNA using a RevertAid First Strand cDNA synthesis kit (www.thermofisher.com/). Primers were designed to be specific to each transcript of interest using Primer 3 (ref. ^92^) to generate PCR products between 100 and 200 bp in length. The qPCR mixture consisted of 10 μl of 2× SensiFAST SYBR No-ROX mastermix (www.meridianbioscience.com/), 400 nM forward and reverse primers, 1 μl of diluted (1 in 4) cDNA and sterile water in a total volume of 20 μl. The qPCRs were performed in triplicate on a CFX Connect thermocycler (www.bio-rad.com) with the following cycling conditions: 95 °C for 2 min, followed by 40 cycles of 95 °C for 10 s and 60 °C for 30 s. A melting curve step (50–95 °C) was then performed to ensure that only a single product had been amplified in each reaction. Standard curves were performed for each primer pair with a dilution series of cDNA to calculate primer efficiencies. By plotting quantification of cycle (Cq) values against the log_10_ of the different dilutions, PCR efficiency was calculated as *E* = 10^(−1/slope)^ − 1, using the BioRad CFX Connect thermocycler software. ‘No template’ and ‘no reverse transcriptase’ controls were also performed for each primer pair and cDNA, respectively. To normalize the gene expression data, the RefFinder web-tool^93^ was used to select the most stable reference gene from two candidates: *ACTIN2* (*ACT2*) and *UBIQUITIN5* (*UBQ5*). *ACT2* was the most stable and was used to normalize the data. Relative mean expression levels were calculated using the 2^−ΔΔCq^ method following normalization against *ACT2*. Statistical analysis was performed on normalized ΔΔCq values relative to the mean for the wild type using Welch’s *t*-test.

### Reporting summary

Further information on research design is available in the Nature Portfolio Reporting Summary linked to this article.

## Supplementary information

Supplementary Information Supplementary Figs. 1 and 2.

Reporting Summary

Supplementary Tables 1–5 Tables of primer sequences, metaphase I chromosome area measurements, chiasma counts and ZYP1C/ZYP1N foci counts.

## Source data

Source Data Figs. 1 and 3 and Extended Data Fig. 1 Statistical source data for Figs. 1x,y,c′,d′ and 3c and Extended Data Fig. 1b,c.

## Data Availability

The SCEP3 mRNA complete coding sequence has been deposited in GenBank (PP962290.1). Source data are provided with this paper. All other data supporting the findings of this research are presented in the main text, figures and Supplementary Information.
