# Supplementary material for: SCEP3 initiates synapsis and implements crossover interference in Arabidopsis
Source: Nat Plants. 2025 Nov 18;11(12):2531–47. doi: 10.1038/s41477-025-02155-x (PMC12711579; doi:10.1038/s41477-025-02155-x)
Supplement: Supplementary file 1 — Supplementary Figs. 1 and 2. [file 41477_2025_2155_MOESM1_ESM.pdf]

nature plants

Supplementary information

https://doi.org/10.1038/s41477-025-02155-x

SCEP3 initiates synapsis and implements crossover interference in *Arabidopsis*

In the format provided by the authors and unedited

scep3-2$^{-/-}$

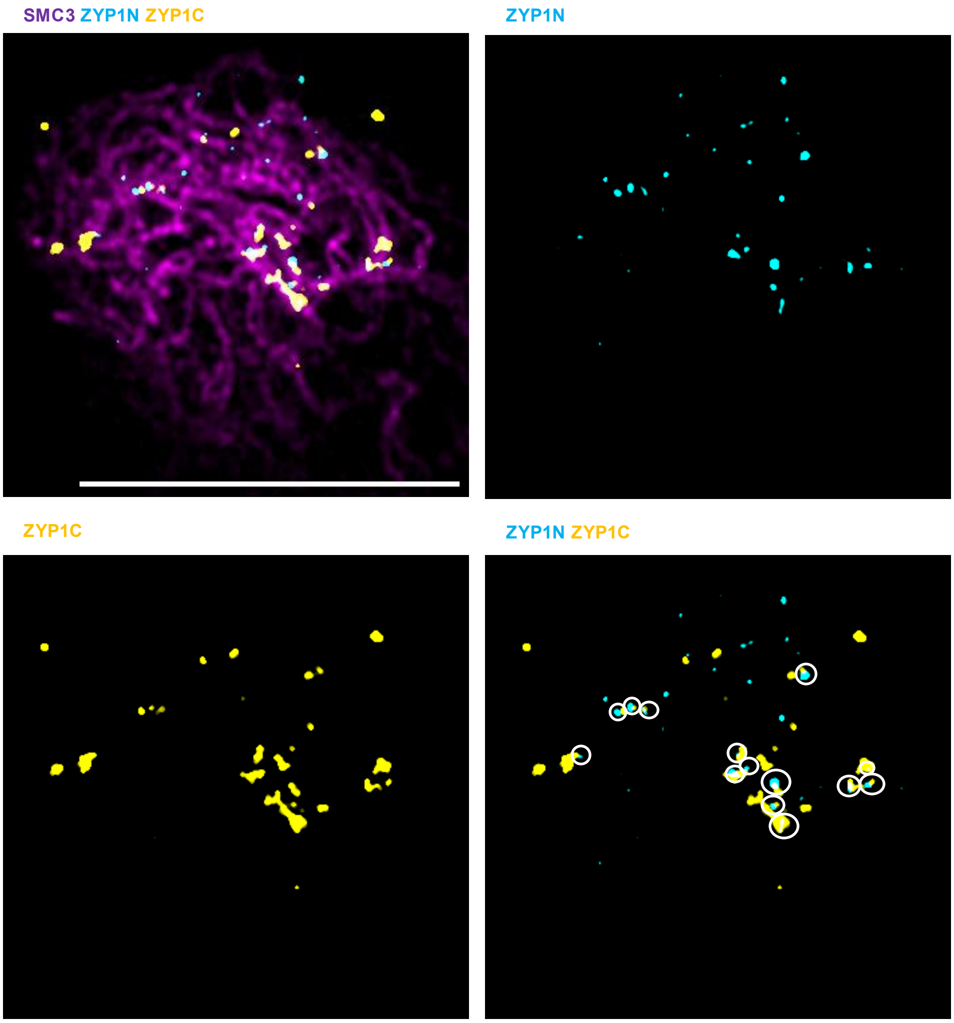

Supplementary Fig. 1. In the *scep3-2* homozygous mutant ZYP1 signal loads as foci that do not polymerise. Co-immunofluorescence of ZYP1C (yellow) and ZYP1N (blue) with SMC3 (magenta) reveals co-localisation of the larger foci (shown by white circles) indicating that they are ZYP1 molecules and not background. Scale bar = 10µm.

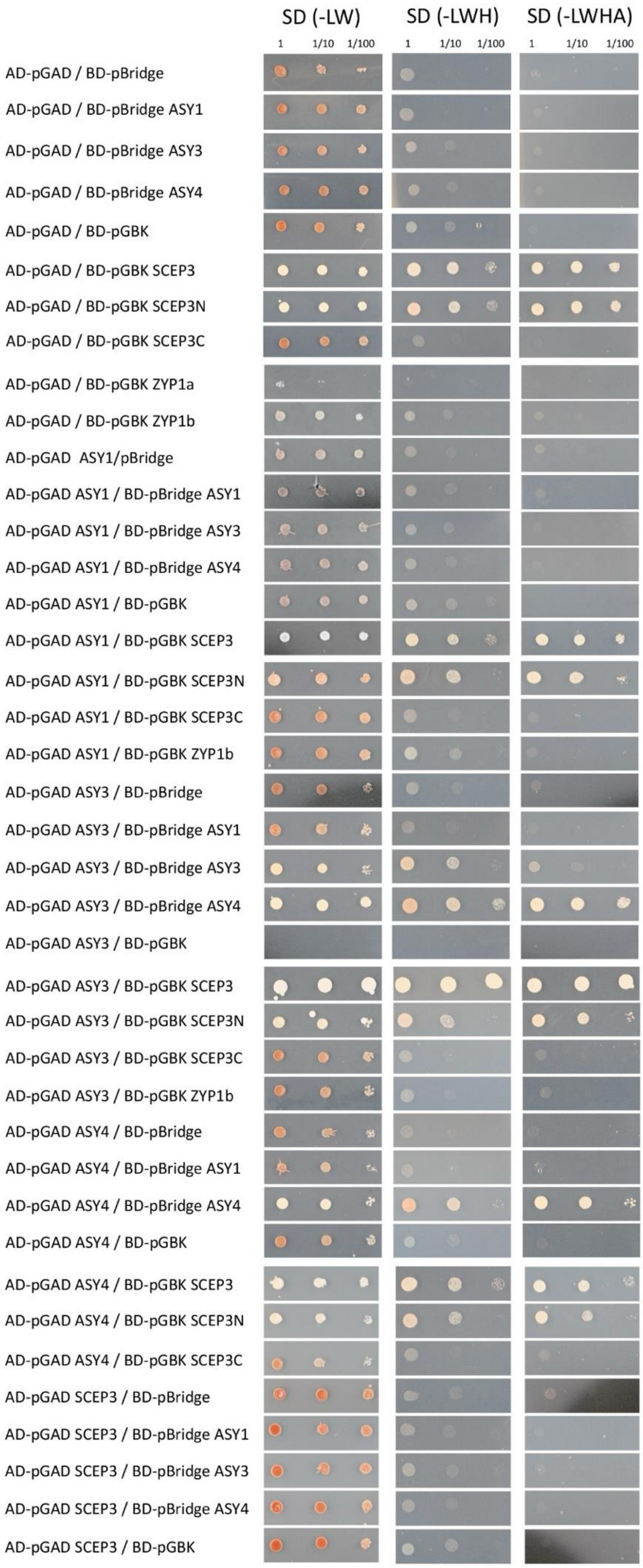
SD (-LW)
SD (-LWH)
SD (-LWHA)
1
1/10
1/100
AD-pGAD / BD-pBridge
AD-pGAD / BD-pBridge ASY1
AD-pGAD / BD-pBridge ASY3
AD-pGAD / BD-pBridge ASY4
AD-pGAD / BD-pGBK
AD-pGAD / BD-pGBK SCEP3
AD-pGAD / BD-pGBK SCEP3N
AD-pGAD / BD-pGBK SCEP3C
AD-pGAD / BD-pGBK ZYP1a
AD-pGAD / BD-pGBK ZYP1b
AD-pGAD ASY1/pBridge
AD-pGAD ASY1 / BD-pBridge ASY1
AD-pGAD ASY1 / BD-pBridge ASY3
AD-pGAD ASY1 / BD-pBridge ASY4
AD-pGAD ASY1 / BD-pGBK
AD-pGAD ASY1 / BD-pGBK SCEP3
AD-pGAD ASY1 / BD-pGBK SCEP3N
AD-pGAD ASY1 / BD-pGBK SCEP3C
AD-pGAD ASY1 / BD-pGBK ZYP1b
AD-pGAD ASY3 / BD-pBridge
AD-pGAD ASY3 / BD-pBridge ASY1
AD-pGAD ASY3 / BD-pBridge ASY3
AD-pGAD ASY3 / BD-pBridge ASY4
AD-pGAD ASY3 / BD-pGBK
AD-pGAD ASY3 / BD-pGBK SCEP3
AD-pGAD ASY3 / BD-pGBK SCEP3N
AD-pGAD ASY3 / BD-pGBK SCEP3C
AD-pGAD ASY3 / BD-pGBK ZYP1b
AD-pGAD ASY4 / BD-pBridge
AD-pGAD ASY4 / BD-pBridge ASY1
AD-pGAD ASY4 / BD-pBridge ASY4
AD-pGAD ASY4 / BD-pGBK
AD-pGAD ASY4 / BD-pGBK SCEP3
AD-pGAD ASY4 / BD-pGBK SCEP3N
AD-pGAD ASY4 / BD-pGBK SCEP3C
AD-pGAD SCEP3 / BD-pBridge
AD-pGAD SCEP3 / BD-pBridge ASY1
AD-pGAD SCEP3 / BD-pBridge ASY3
AD-pGAD SCEP3 / BD-pBridge ASY4
AD-pGAD SCEP3 / BD-pGBK

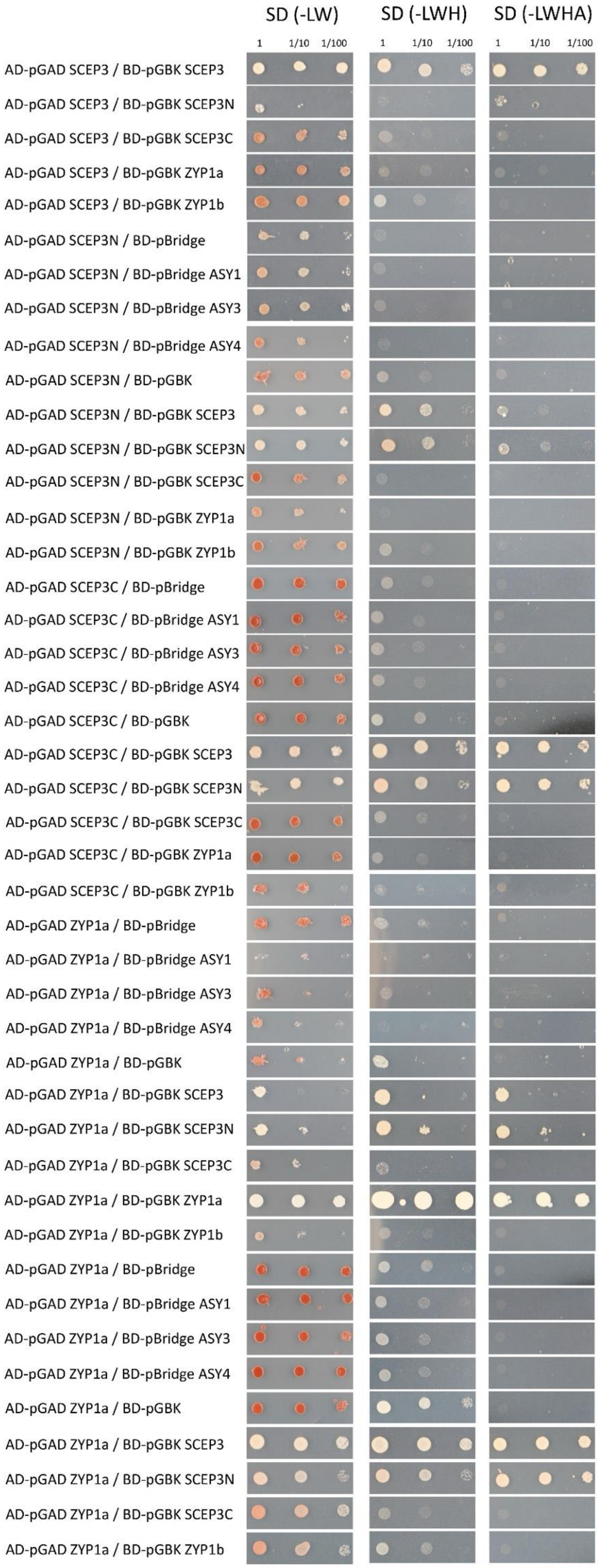
SD (-LW)
SD (-LWH)
SD (-LWHA)
1
1/10
1/100
AD-pGAD SCEP3 / BD-pGBK SCEP3
AD-pGAD SCEP3 / BD-pGBK SCEP3N
AD-pGAD SCEP3 / BD-pGBK SCEP3C
AD-pGAD SCEP3 / BD-pGBK ZYP1a
AD-pGAD SCEP3 / BD-pGBK ZYP1b
AD-pGAD SCEP3N / BD-pBridge
AD-pGAD SCEP3N / BD-pBridge ASY1
AD-pGAD SCEP3N / BD-pBridge ASY3
AD-pGAD SCEP3N / BD-pBridge ASY4
AD-pGAD SCEP3N / BD-pGBK
AD-pGAD SCEP3N / BD-pGBK SCEP3
AD-pGAD SCEP3N / BD-pGBK SCEP3N
AD-pGAD SCEP3N / BD-pGBK SCEP3C
AD-pGAD SCEP3N / BD-pGBK ZYP1a
AD-pGAD SCEP3N / BD-pGBK ZYP1b
AD-pGAD SCEP3C / BD-pBridge
AD-pGAD SCEP3C / BD-pBridge ASY1
AD-pGAD SCEP3C / BD-pBridge ASY3
AD-pGAD SCEP3C / BD-pBridge ASY4
AD-pGAD SCEP3C / BD-pGBK
AD-pGAD SCEP3C / BD-pGBK SCEP3
AD-pGAD SCEP3C / BD-pGBK SCEP3N
AD-pGAD SCEP3C / BD-pGBK SCEP3C
AD-pGAD SCEP3C / BD-pGBK ZYP1a
AD-pGAD SCEP3C / BD-pGBK ZYP1b
AD-pGAD ZYP1a / BD-pBridge
AD-pGAD ZYP1a / BD-pBridge ASY1
AD-pGAD ZYP1a / BD-pBridge ASY3
AD-pGAD ZYP1a / BD-pBridge ASY4
AD-pGAD ZYP1a / BD-pGBK
AD-pGAD ZYP1a / BD-pGBK SCEP3
AD-pGAD ZYP1a / BD-pGBK SCEP3N
AD-pGAD ZYP1a / BD-pGBK SCEP3C
AD-pGAD ZYP1a / BD-pGBK ZYP1a
AD-pGAD ZYP1a / BD-pGBK ZYP1b
AD-pGAD ZYP1a / BD-pBridge
AD-pGAD ZYP1a / BD-pBridge ASY1
AD-pGAD ZYP1a / BD-pBridge ASY3
AD-pGAD ZYP1a / BD-pBridge ASY4
AD-pGAD ZYP1a / BD-pGBK
AD-pGAD ZYP1a / BD-pGBK SCEP3
AD-pGAD ZYP1a / BD-pGBK SCEP3N
AD-pGAD ZYP1a / BD-pGBK SCEP3C
AD-pGAD ZYP1a / BD-pGBK ZYP1b

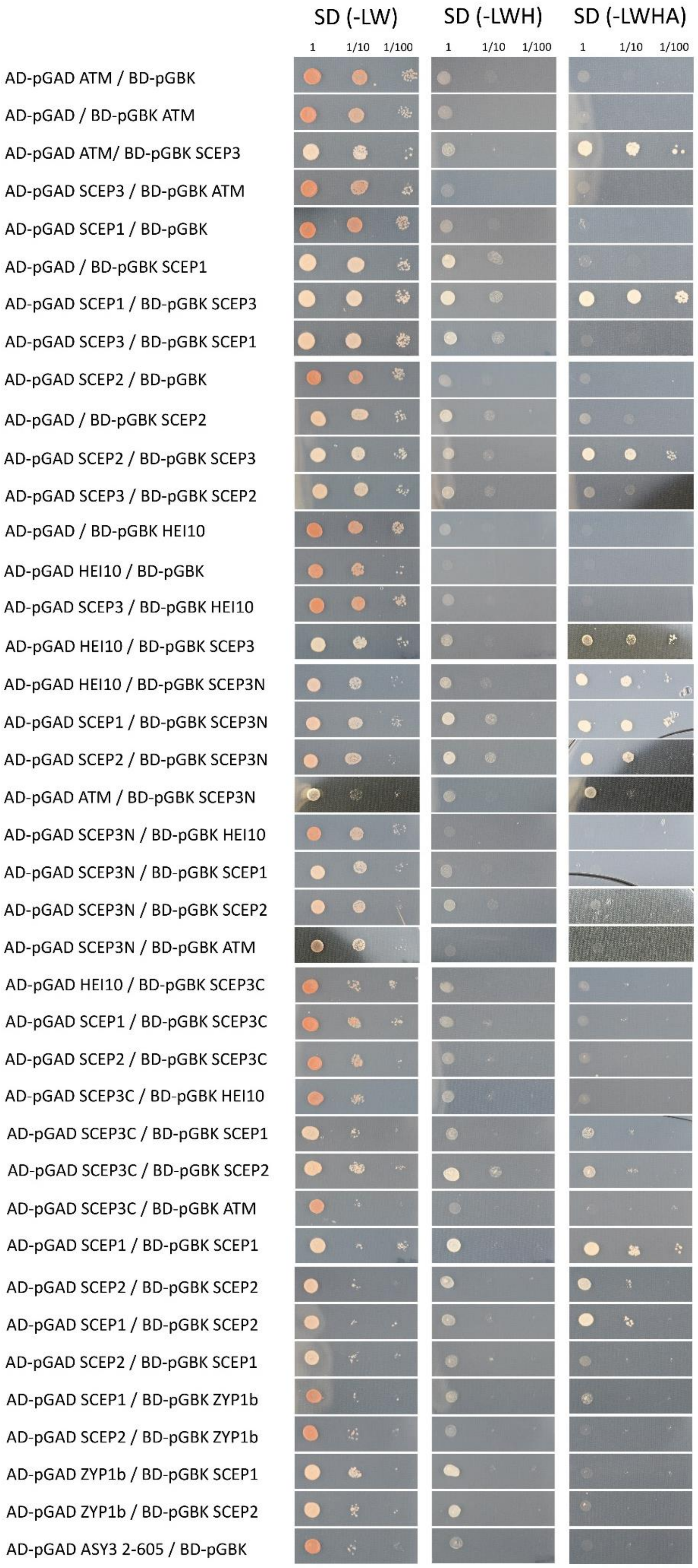
SD (-LW)
SD (-LWH)
SD (-LWHA)
1
1/10
1/100
AD-pGAD ATM / BD-pGBK
AD-pGAD / BD-pGBK ATM
AD-pGAD ATM/ BD-pGBK SCEP3
AD-pGAD SCEP3 / BD-pGBK ATM
AD-pGAD SCEP1 / BD-pGBK
AD-pGAD / BD-pGBK SCEP1
AD-pGAD SCEP1 / BD-pGBK SCEP3
AD-pGAD SCEP3 / BD-pGBK SCEP1
AD-pGAD SCEP2 / BD-pGBK
AD-pGAD / BD-pGBK SCEP2
AD-pGAD SCEP2 / BD-pGBK SCEP3
AD-pGAD SCEP3 / BD-pGBK SCEP2
AD-pGAD / BD-pGBK HEI10
AD-pGAD HEI10 / BD-pGBK
AD-pGAD SCEP3 / BD-pGBK HEI10
AD-pGAD HEI10 / BD-pGBK SCEP3
AD-pGAD HEI10 / BD-pGBK SCEP3N
AD-pGAD SCEP1 / BD-pGBK SCEP3N
AD-pGAD SCEP2 / BD-pGBK SCEP3N
AD-pGAD ATM / BD-pGBK SCEP3N
AD-pGAD SCEP3N / BD-pGBK HEI10
AD-pGAD SCEP3N / BD-pGBK SCEP1
AD-pGAD SCEP3N / BD-pGBK SCEP2
AD-pGAD SCEP3N / BD-pGBK ATM
AD-pGAD HEI10 / BD-pGBK SCEP3C
AD-pGAD SCEP1 / BD-pGBK SCEP3C
AD-pGAD SCEP2 / BD-pGBK SCEP3C
AD-pGAD SCEP3C / BD-pGBK HEI10
AD-pGAD SCEP3C / BD-pGBK SCEP1
AD-pGAD SCEP3C / BD-pGBK SCEP2
AD-pGAD SCEP3C / BD-pGBK ATM
AD-pGAD SCEP1 / BD-pGBK SCEP1
AD-pGAD SCEP2 / BD-pGBK SCEP2
AD-pGAD SCEP1 / BD-pGBK SCEP2
AD-pGAD SCEP2 / BD-pGBK SCEP1
AD-pGAD SCEP1 / BD-pGBK ZYP1b
AD-pGAD SCEP2 / BD-pGBK ZYP1b
AD-pGAD ZYP1b / BD-pGBK SCEP1
AD-pGAD ZYP1b / BD-pGBK SCEP2
AD-pGAD ASY3 2-605 / BD-pGBK

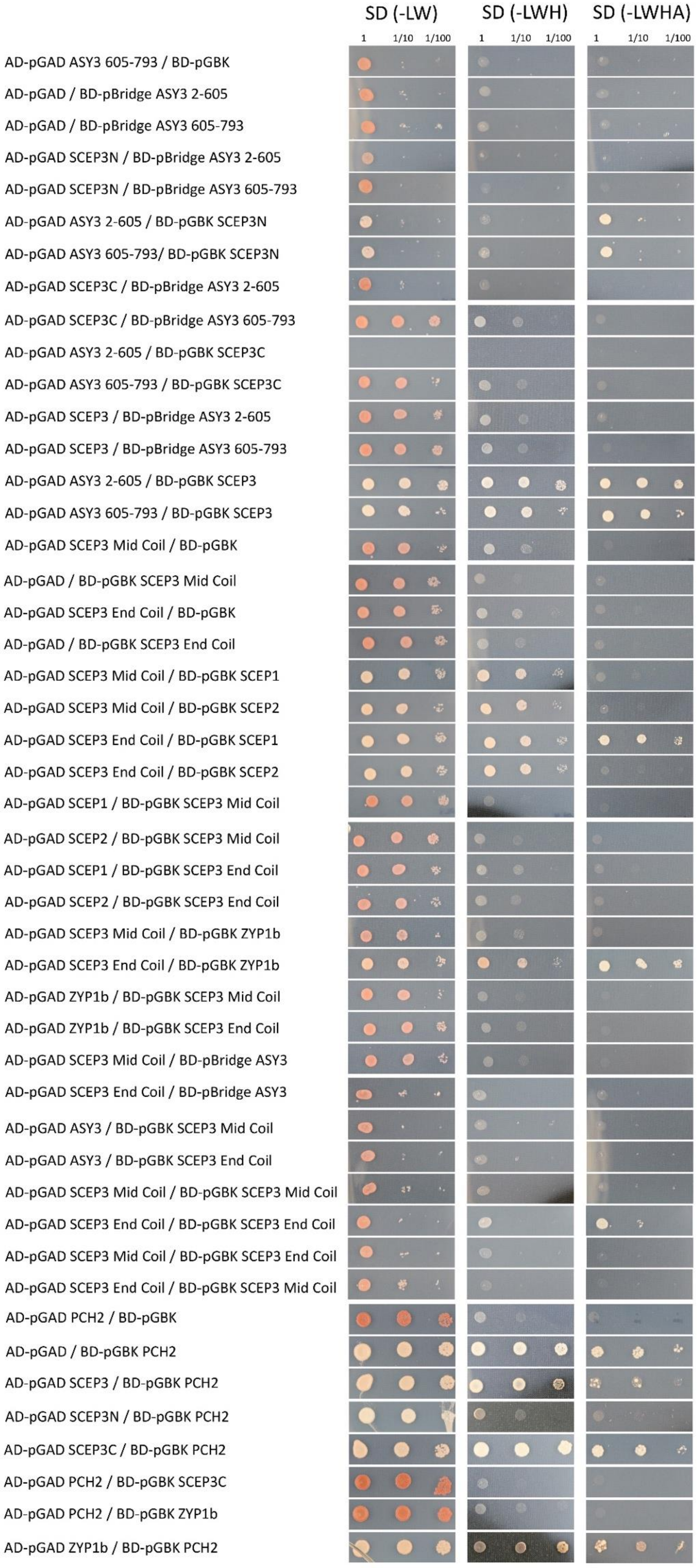
SD (-LW)
SD (-LWH)
SD (-LWHA)
1
1/10
1/100
AD-pGAD ASY3 605-793 / BD-pGBK
AD-pGAD / BD-pBridge ASY3 2-605
AD-pGAD / BD-pBridge ASY3 605-793
AD-pGAD SCEP3N / BD-pBridge ASY3 2-605
AD-pGAD SCEP3N / BD-pBridge ASY3 605-793
AD-pGAD ASY3 2-605 / BD-pGBK SCEP3N
AD-pGAD ASY3 605-793/ BD-pGBK SCEP3N
AD-pGAD SCEP3C / BD-pBridge ASY3 2-605
AD-pGAD SCEP3C / BD-pBridge ASY3 605-793
AD-pGAD ASY3 2-605 / BD-pGBK SCEP3C
AD-pGAD ASY3 605-793 / BD-pGBK SCEP3C
AD-pGAD SCEP3 / BD-pBridge ASY3 2-605
AD-pGAD SCEP3 / BD-pBridge ASY3 605-793
AD-pGAD ASY3 2-605 / BD-pGBK SCEP3
AD-pGAD ASY3 605-793 / BD-pGBK SCEP3
AD-pGAD SCEP3 Mid Coil / BD-pGBK
AD-pGAD / BD-pGBK SCEP3 Mid Coil
AD-pGAD SCEP3 End Coil / BD-pGBK
AD-pGAD / BD-pGBK SCEP3 End Coil
AD-pGAD SCEP3 Mid Coil / BD-pGBK SCEP1
AD-pGAD SCEP3 Mid Coil / BD-pGBK SCEP2
AD-pGAD SCEP3 End Coil / BD-pGBK SCEP1
AD-pGAD SCEP3 End Coil / BD-pGBK SCEP2
AD-pGAD SCEP1 / BD-pGBK SCEP3 Mid Coil
AD-pGAD SCEP2 / BD-pGBK SCEP3 Mid Coil
AD-pGAD SCEP1 / BD-pGBK SCEP3 End Coil
AD-pGAD SCEP2 / BD-pGBK SCEP3 End Coil
AD-pGAD SCEP3 Mid Coil / BD-pGBK ZYP1b
AD-pGAD SCEP3 End Coil / BD-pGBK ZYP1b
AD-pGAD ZYP1b / BD-pGBK SCEP3 Mid Coil
AD-pGAD ZYP1b / BD-pGBK SCEP3 End Coil
AD-pGAD SCEP3 Mid Coil / BD-pBridge ASY3
AD-pGAD SCEP3 End Coil / BD-pBridge ASY3
AD-pGAD ASY3 / BD-pGBK SCEP3 Mid Coil
AD-pGAD ASY3 / BD-pGBK SCEP3 End Coil
AD-pGAD SCEP3 Mid Coil / BD-pGBK SCEP3 Mid Coil
AD-pGAD SCEP3 End Coil / BD-pGBK SCEP3 End Coil
AD-pGAD SCEP3 Mid Coil / BD-pGBK SCEP3 End Coil
AD-pGAD SCEP3 End Coil / BD-pGBK SCEP3 Mid Coil
AD-pGAD PCH2 / BD-pGBK
AD-pGAD / BD-pGBK PCH2
AD-pGAD SCEP3 / BD-pGBK PCH2
AD-pGAD SCEP3N / BD-pGBK PCH2
AD-pGAD SCEP3C / BD-pGBK PCH2
AD-pGAD PCH2 / BD-pGBK SCEP3C
AD-pGAD PCH2 / BD-pGBK ZYP1b
AD-pGAD ZYP1b / BD-pGBK PCH2

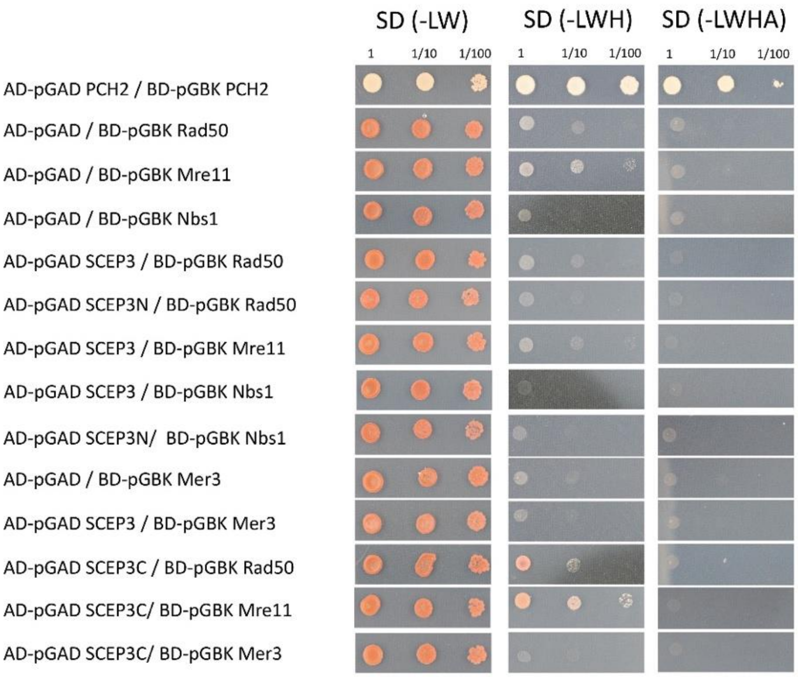

Supplementary Fig. 2. SCEP3 strongly interacts with SC proteins in Yeast-2-Hybrid assays. Synthetic amino acid double dropout (DDO-LW -Leucine, -Tryptophan), triple dropout (TDO-LWH - Leucine, -Tryptophan, -Histidine) and quadruple dropout (QDO-LWHA - Leucine, -Tryptophan, -Histidine, -Adenine) media showing growth of yeast with controls and tested gene combinations spotted in serial dilutions (Undiluted, 1/10 dilution and 1/100 dilution) and grown for ~72 hours at 30°C. AD = activation domain, BD = binding domain.
