# Supplementary material for: SCEP3 initiates synapsis and implements crossover interference in Arabidopsis
Source: Nat Plants. 2025 Nov 18;11(12):2531–47. doi: 10.1038/s41477-025-02155-x (PMC12711579; doi:10.1038/s41477-025-02155-x)
Supplement: Supplementary file 2 — Reporting Summary [file 41477_2025_2155_MOESM2_ESM.pdf]

nature portfolio

Corresponding author(s): James D Higgins

Last updated by author(s): 03/10//2025

Reporting Summary

Nature Portfolio wishes to improve the reproducibility of the work that we publish. This form provides structure for consistency and transparency in reporting. For further information on Nature Portfolio policies, see our Editorial Policies and the Editorial Policy Checklist.

Statistics

For all statistical analyses, confirm that the following items are present in the figure legend, table legend, main text, or Methods section.

n/a	Confirmed	
☐	☒	The exact sample size (*n*) for each experimental group/condition, given as a discrete number and unit of measurement
☐	☒	A statement on whether measurements were taken from distinct samples or whether the same sample was measured repeatedly
☐	☒	The statistical test(s) used AND whether they are one- or two-sided<br>*Only common tests should be described solely by name; describe more complex techniques in the Methods section.*
☒	☐	A description of all covariates tested
☒	☐	A description of any assumptions or corrections, such as tests of normality and adjustment for multiple comparisons
☐	☒	A full description of the statistical parameters including central tendency (e.g. means) or other basic estimates (e.g. regression coefficient) AND variation (e.g. standard deviation) or associated estimates of uncertainty (e.g. confidence intervals)
☐	☒	For null hypothesis testing, the test statistic (e.g. *F*, *t*, *r*) with confidence intervals, effect sizes, degrees of freedom and *P* value noted<br>*Give P values as exact values whenever suitable.*
☒	☐	For Bayesian analysis, information on the choice of priors and Markov chain Monte Carlo settings
☒	☐	For hierarchical and complex designs, identification of the appropriate level for tests and full reporting of outcomes
☒	☐	Estimates of effect sizes (e.g. Cohen's *d*, Pearson's *r*), indicating how they were calculated

Our web collection on statistics for biologists contains articles on many of the points above.

Software and code

Policy information about availability of computer code

Data collection	Fluorescence images were acquired using a Nikon Eclipse Ni-E microscope (Nikon, Tokyo, Japan) equipped with a Nikon DS-Qi1Mc camera or a Nikon Eclipse Ci microscope equipped with a Photometrics CoolSNAP DYNO camera and processed using NIS-Elements AR version 4.6. Super-resolution fluorescence images were acquired with the Zeiss LSM 980 Airyscan 2 (Carl Zeiss GmbH) and ZEN2.3 (blue edition) using default Airyscan processing in the Advanced Imaging Facility at University of Leicester and processed using ImageJ (Version 2.0/1.52).
Data analysis	Statistical analysis: R (4.4.1) with fitdist function from the fitdistrplus R package fro CO analysis; GraphPad Prism (10.4.0); MiniTab (21.2); Microsoft Excel (16.91).<br>Sequence alignment: Geneious (11.1.5). FTL interference ratios: Stahl Lab Online tools (https://elizabethhousworth.com/StahlLabOnlineTools/<br>Protein structure: https://www.sbg.bio.ic.ac.uk/phyre2/html/page.cgi?id=index, https://cb.csail.mit.edu/cb/multicoil2/cgi-bin/multicoil2.cgi, https://alphafoldserver.com/

For manuscripts utilizing custom algorithms or software that are central to the research but not yet described in published literature, software must be made available to editors and reviewers. We strongly encourage code deposition in a community repository (e.g. GitHub). See the Nature Portfolio guidelines for submitting code & software for further information.

Data

Policy information about availability of data

All manuscripts must include a data availability statement. This statement should provide the following information, where applicable:

- Accession codes, unique identifiers, or web links for publicly available datasets
- A description of any restrictions on data availability
- For clinical datasets or third party data, please ensure that the statement adheres to our policy

All data supporting the findings of this work are available in the main text and supplementary information files. Sequence data is freely available in GenBank (https://www.ncbi.nlm.nih.gov/genbank/) under the accession number PP962290. All protein sequences can be found in UNIPROT (https://www.uniprot.org/) and Genbank.

Research involving human participants, their data, or biological material

Policy information about studies with human participants or human data. See also policy information about sex, gender (identity/presentation), and sexual orientation and race, ethnicity and racism.

Reporting on sex and gender	n/a
Reporting on race, ethnicity, or other socially relevant groupings	*Please specify the socially constructed or socially relevant categorization variable(s) used in your manuscript and explain why they were used. Please note that such variables should not be used as proxies for other socially constructed/relevant variables (for example, race or ethnicity should not be used as a proxy for socioeconomic status).*<br>*Provide clear definitions of the relevant terms used, how they were provided (by the participants/respondents, the researchers, or third parties), and the method(s) used to classify people into the different categories (e.g. self-report, census or administrative data, social media data, etc.)*<br>*Please provide details about how you controlled for confounding variables in your analyses.*
Population characteristics	*Describe the covariate-relevant population characteristics of the human research participants (e.g. age, genotypic information, past and current diagnosis and treatment categories). If you filled out the behavioural & social sciences study design questions and have nothing to add here, write "See above."*
Recruitment	*Describe how participants were recruited. Outline any potential self-selection bias or other biases that may be present and how these are likely to impact results.*
Ethics oversight	*Identify the organization(s) that approved the study protocol.*

Note that full information on the approval of the study protocol must also be provided in the manuscript.

Field-specific reporting

Please select the one below that is the best fit for your research. If you are not sure, read the appropriate sections before making your selection.

☒ Life sciences ☐ Behavioural & social sciences ☐ Ecological, evolutionary & environmental sciences

For a reference copy of the document with all sections, see nature.com/documents/nr-reporting-summary-flat.pdf

Life sciences study design

All studies must disclose on these points even when the disclosure is negative.

Sample size	No sample size calculations were performed. Sample sizes were based on published work of similar experiments and were sufficient to result in statistical significance.
Data exclusions	No data were excluded.
Replication	All data were reproducible, and numbers of replicates are stated in the main text and supplementary information.
Randomization	Plants of different genotypes were randomized and grown under controlled conditions (constant 22C, 65% relative humidity, and a 16h/8h light/dark photoperiod) to avoid environmental effects.
Blinding	Blinding was not relevant to our study as there was no prior knowledge of the experimental outcomes.

Reporting for specific materials, systems and methods

We require information from authors about some types of materials, experimental systems and methods used in many studies. Here, indicate whether each material, system or method listed is relevant to your study. If you are not sure if a list item applies to your research, read the appropriate section before selecting a response.

Materials & experimental systems

n/a	Involved in the study
☐	☒ Antibodies
☒	☐ Eukaryotic cell lines
☒	☐ Palaeontology and archaeology
☒	☐ Animals and other organisms
☒	☐ Clinical data
☒	☐ Dual use research of concern
☐	☒ Plants

Methods

n/a	Involved in the study
☒	☐ ChIP-seq
☒	☐ Flow cytometry
☒	☐ MRI-based neuroimaging

Antibodies

Antibodies used

proteins were stained using the following primary antibodies at a concentration of 1:500: rat α-AtZYP1-C, rat/guinea pig α-AtZYP1-C, rabbit α-AtZYP1-N, guinea pig/rat α-ASY1, rabbit α-ASY3, rabbit α-PCH2, rabbit α-REC8, rat α-SCEP3N and rabbit α-SCEP3C.

Validation

Validation of antibodies was performed in previous publications except for SCEP3N and SCEP3C that were validated in this study.

Plants

Seed stocks

scep3-2 (SALK_098044), scep3-4, scep3-5 (SALK_023936), scep3-6 (GK500E03), asy1-4 (SALK_046272), asy3-1 (SALK_143676), asy4-3, pch2-1 (SAIL_1187_C06), msh5-1 (SALK_110240), dmc1 (SAIL_170_F08), spo11-1-5 (SALK_131704) and hei10-2 (SALK_014624) were acquired from the Nottingham Arabidopsis Stock Centre (https://arabidopsis.info/) and zyp1a-2 was previously generated.

Novel plant genotypes

Describe the methods by which all novel plant genotypes were produced. This includes those generated by transgenic approaches, gene editing, chemical/radiation-based mutagenesis and hybridization. For transgenic lines, describe the transformation method, the number of independent lines analyzed and the generation upon which experiments were performed. For gene-edited lines, describe the editor used, the endogenous sequence targeted for editing, the targeting guide RNA sequence (if applicable) and how the editor was applied.

Authentication

All lines were previously published except for spo11-1-5 (SALK_131704) that we validated to be phenotypically consistent with previous null alleles of spo11-1.
